# Regulation of glial size by eicosapentaenoic acid through a novel Golgi apparatus mechanism

**DOI:** 10.1371/journal.pbio.3001051

**Published:** 2020-12-28

**Authors:** Albert Zhang, Ziqiang Guan, Kyle Ockerman, Pengyuan Dong, Jiansheng Guo, Zhiping Wang, Dong Yan

**Affiliations:** 1 Department of Molecular Genetics and Microbiology, Duke University Medical Center, Durham, North Carolina, United States of America; 2 Department of Biochemistry, Duke University Medical Center, Durham, North Carolina, United States of America; 3 Center of Cryo-Electron Microscopy, Zhejiang University, Hangzhou, China; 4 Institute of Neuroscience and Department of Neurology of Second Affiliated Hospital, NHC and CAMS Key Laboratory of Medical Neurobiology, Zhejiang University School of Medicine, Hangzhou, China; 5 Department of Neurobiology, Regeneration Next Initiative, and Duke Institute for Brain Sciences, Duke University Medical Center, Durham, North Carolina, United States of America; UCSD, UNITED STATES

## Abstract

Coordination of cell growth is essential for the development of the brain, but the molecular mechanisms underlying the regulation of glial and neuronal size are poorly understood. To investigate the mechanisms involved in glial size regulation, we used *Caenorhabditis elegans* amphid sheath (AMsh) glia as a model and show that a conserved *cis*-Golgi membrane protein *eas-1/GOLT1B* negatively regulates glial growth. We found that *eas-1* inhibits a conserved E3 ubiquitin ligase *rnf-145*/RNF145, which, in turn, promotes nuclear activation of *sbp-1/* SREBP, a key regulator of sterol and fatty acid synthesis, to restrict cell growth. At early developmental stages, *rnf-145* in the *cis*-Golgi network inhibits *sbp-1* activation to promote the growth of glia, and when animals reach the adult stage, this inhibition is released through an *eas-1-*dependent shuttling of *rnf-145* from the *cis*-Golgi to the *trans*-Golgi network to stop glial growth. Furthermore, we identified long-chain polyunsaturated fatty acids (LC-PUFAs), especially eicosapentaenoic acid (EPA), as downstream products of the *eas-1-rnf-145-sbp-1* pathway that functions to prevent the overgrowth of glia. Together, our findings reveal a novel and potentially conserved mechanism underlying glial size control.

## Abstract 2

The molecular mechanisms underlying the regulation of glial and neuronal size are poorly understood. This study in nematodes reveals eicosapentaenoic acid as the downstream product of a pathway that functions to prevent the overgrowth of glia, suggesting a novel and potentially conserved mechanism underlying glial size control.

## Introduction

A long-standing question in biology is how cells regulate their size [1–3]. In unicellular organisms such as yeast, the extracellular nutritional environment plays a key role in controlling cell size, and this process is tightly coupled with cell proliferation [2,4,5]. On the other hand, multicellular organisms possess many terminally differentiated cells like neurons and glia that use different mechanisms to regulate their sizes from dividing cells [2].

In the nervous system, morphologically distinct glial cells grow and form close interactions with other cells [6–8]. The size of glial cells needs to be tightly controlled as they will not be able to properly interact with other cells if undersized and occupy extra space to prevent the growth of other cells if oversized. However, there is still much unknown about the underlying mechanisms controlling glial size, especially given the diversity of cellular contexts.

To study molecular mechanisms involved in glial size control, we used genetic and imaging techniques to study *Caenorhabditis elegans* glia. Of the 56 glia in the worm, we focused in particular on a pair of terminally differentiated glia in *C*. *elegans* called the amphid sheath (AMsh) glia, which comprises the largest chemosensory organ in the worm and whose function is among the most well studied [9–11]. Each AMsh glia ensheathes the dendritic tips of 12 sensory neurons in head, which requires precise regulation of their morphology [12]. In addition, AMsh glia are essential for the proper function of many of the neurons it ensheathes [11], allowing for the study of potential functional impacts of glial size disruption. We started with an unbiased forward genetic screen for genes that affect AMsh cell size, and uncovered the function of a previously uncharacterized *C*. *elegans* gene, *eas-1 (*enlarged amphid sheath), in regulating AMsh cell size.

*eas-1* is a homolog of human Golgi transport B (GOLT1B), a highly conserved membrane protein from yeast to human [13,14]. The studies of GOLT1B were only carried out in yeast and rice, and they showed that its ortholog, Got1, likely functions in the early cisternae of the Golgi complex in addition to the ER exit site (ERES) to facilitate anterograde ER–Golgi transport [13–15]. However, the localization and function of GOLT1B in animals are still completely unknown.

The sterol regulatory element-binding protein (SREBP) family is comprised of basic helix-loop-helix (bHLH) leucine zipper transcriptional factors and have been shown to play key roles in the regulation of lipid homeostasis through regulating the expression of a wide range of enzymes involved in fatty acid and cholesterol synthesis [16,17]. Unlike mammals, *C*. *elegans* are cholesterol auxotrophs [18]. Thus, the single *C*. *elegans* SREBP homolog, SBP-1, is only involved in the regulation of fatty acids but not cholesterol synthesis, where it controls the expression of several lipogenic enzymes [19–21]. Recent studies in the mouse liver demonstrate that a newly identified E3 ubiquitin ligase, RING finger protein 145 (RNF145), can negatively regulate the activation of SREBP-2 in response to the activation of liver X receptor (LXR) and elevated sterol levels [16,22–24]. It is still unclear whether and how the RNF145-SREBP pathway affects other cell types.

We show that *eas-1* can negatively regulate the conserved E3 ubiquitin ligase *rnf-145*/RNF145 and in turn activate *sbp-1/* SREBP to limit the growth of AMsh cells. Furthermore, we find that the long-chain polyunsaturated fatty acid (LC-PUFAs) eicosapentaenoic acid (EPA) is likely a downstream product of this pathway that helps to set the brakes on cell growth in AMsh glia. Our findings uncover a novel and potentially conserved pathway regulating glial size and represents the first reported function of *eas-1/*GOLT1B in animals.

## Results

### *eas-1* functions cell autonomously to regulate glial size

**Fig 1:**
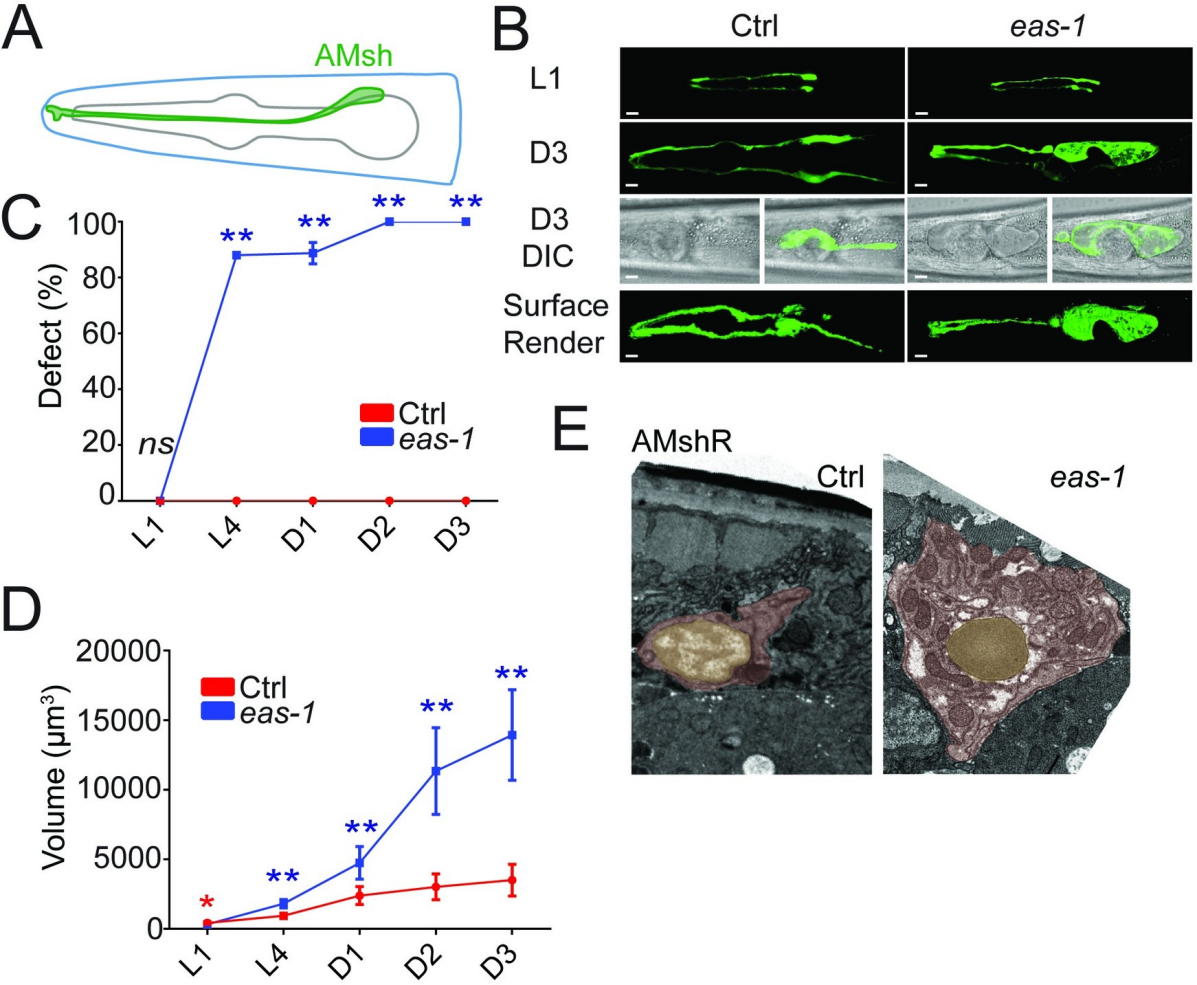
*eas-1* regulates the size of AMsh cells. (**A**) Schematic representation of an AMsh cell labeled in green. (**B**) Confocal images of AMsh cells from L1 and D3 adult stages in WT and *eas-1(yad70)* animals expressing *Pf53f4*.*13*::*GFP* (*yadIs48*). Below are merged fluorescence and Nomarski images of AMsh cells. The final row are surface renders of the AMsh cells using Imaris. (**C**) The percentage of WT (red) and *eas-1(yad70)* (blue) animals with enlarged AMsh cells from L1 to D3 adults. Two-way ANOVA, followed by Tukey HSD test. Each point represents 3 biological replicates of at least 50 worms. (**D**) The volume of AMsh cells in WT (red) and *eas-1(yad70)* (blue) animals in μm^3^ from L1 to D3 adults. During stages with incomplete penetrance such as L4 and D1 adult, only *eas-1(yad70)* animals with the enlarged AMsh phenotype were picked for quantification. Two-way ANOVA, followed by Tukey HSD test. Each point represents at least 10 animals. (**E**) Representative transmission electron micrographs of the AMshR cells in WT (left) and *eas-1(yad70)* (right) animals. The soma is highlighted in red, and the nucleus is highlighted in yellow. Scale bar, 10 μm. Data are represented as mean ± SD. *p < 0.05, **p < 0.01. Underlying data for graphs can be found in S1 Data. AMsh, amphid sheath; AMshR, right amphid sheath; ANOVA, analysis of variance; D1, day 1; D3, day 3; HSD, honestly significant difference; SD, standard deviation; WT, wild-type.

To identify genes that may potentially regulate cell size, we conducted an unbiased forward genetic screen for mutants with abnormally sized AMsh cells and isolated a mutant *yad70* with enlarged AMsh cells (Fig 1A and 1B). *yad70* animals exhibited enlarged AMsh cells that increased in penetrance from larval stages to day 3 (D3) adults, where the phenotype penetrance reached 100% by the day 2 (D2) adult stage (Fig 1C). Furthermore, the volume of AMsh cell bodies progressively increased at a faster rate (Fig 1D), and AMsh cells of *yad70* animals were roughly 4 times the size of their control counterparts by D3 (S1A Fig). The AMsh phenotypes observed were not due to an overall increase in animal size, as the length of *yad70* animals were slightly shorter than that of their control counterparts at D2 (S1B Fig). We also noticed that the *yad70* phenotype did not depend on the nutritional condition of animals, as starvation did not suppress the enlarged AMsh cells (S1C Fig). Similarly, phasmid sheath glia (PHsh) also had an enlarged cell size phenotype.

Unlike their AMsh counterparts, the 12 amphid neurons stained by DiI in *yad70* animals appear to have no noticeable change in size or number (S1D and S1E Fig). As uptake and concentration of the lipophilic dye is believed to require exposed cilia, these neurons likely have at least partially functional cilia [25]. Interestingly, *yad70* animals exhibited reduced chemotaxis toward benzaldehyde and pyrazine (S1F and S1G Fig), a behavior mediated by the AWC and AWA neuron, respectively [26]. Similarly, long-range avoidance of the repulsive odorant 1-octanol, a behavior that is partly mediated by ADL neurons [27], was affected in *yad70* mutants (S1H Fig). This is consistent with previous findings that ablation of AMsh cells leads to reduced AWC-mediated chemotaxis toward benzaldehyde, AWA-mediated chemotaxis toward pyrazine, and ADL-mediated long-range repulsion from 1-octanol [11].

It is possible that the enlarged AMsh phenotype observed in *yad70* animals is due to swelling from an abnormal accumulation of certain organelles or vacuoles. For example, in several different models of neurodegeneration, glia may show vacuolation or enlarged lysosomes stained by the late endosome and lysosome marker LAMP-2 [28–33]. To determine whether these organelles may account for the size change, we expressed the late lysosome and lysosome-related organelle (LRO) marker LMP-1/LAMP-1 [34–36] in the AMsh cells of both control and mutant animals (S2A Fig). LAMP-1 was similarly found to label different kinds of vacuoles in animals that arise from normal development or abnormal vacuolization [37–39]. Furthermore, we also expressed a Rab32 homolog fusion protein, mCherry::GLO-1, which was previously reported to localize in LRO membranes and can be used to label certain vacuoles [36,37,40,41], in the AMsh cells of control and mutant animals (S2B Fig). Neither of these markers were able to account for the significant changes in AMsh size in the mutant worms, suggesting that the enlarged size is unlikely due to vacuolation or increased lysosomes and LROs. To further test this, we also conducted RNA interference (RNAi) knockdown of 2 genes important for LRO formation, *glo-1* and *glo-3* [34,36], and found they neither caused any enlarged AMsh phenotype nor suppressed the *yad70* mutant phenotype (S2C Fig). The RNAi knockdown led to significant reductions in gut granule number and size as observed through gut autofluorescence, suggesting efficient knockdown. Lastly, the use of the CellTrace BODIPY TR methyl ester dye, which labels internal membranes [37,42], and Nile Red, which labels certain fat stores and LROs [43–45], show no significant changes in these structures or large vacuoles that can account for the change in cell volume of *yad70* mutants (S2D and S2E Fig). Finally, transmission electron micrographs also showed a greatly enlarged cell soma without any obviously large vacuoles or organelles in *yad70* animals (Fig 1E; S2F Fig). Together, the data suggest that the increased AMsh volume observed is likely due to an overall increase in size rather than an accumulation of large vacuoles, lysosomes, LROs, or lipid droplets.

After sequencing and mapping, we found that *yad70* alters a previously uncharacterized gene, *F41C3*.*4*, and we named it *eas-1* after its enlarged amphid sheath phenotype. The *yad70* allele has a G to A point mutation at the splice site of the last exon, resulting in a frameshift of the last exon that was confirmed through reverse transcription PCR (RT-PCR) and subsequent sequencing of the product (S3A–S3C Fig).

**Fig 2:**
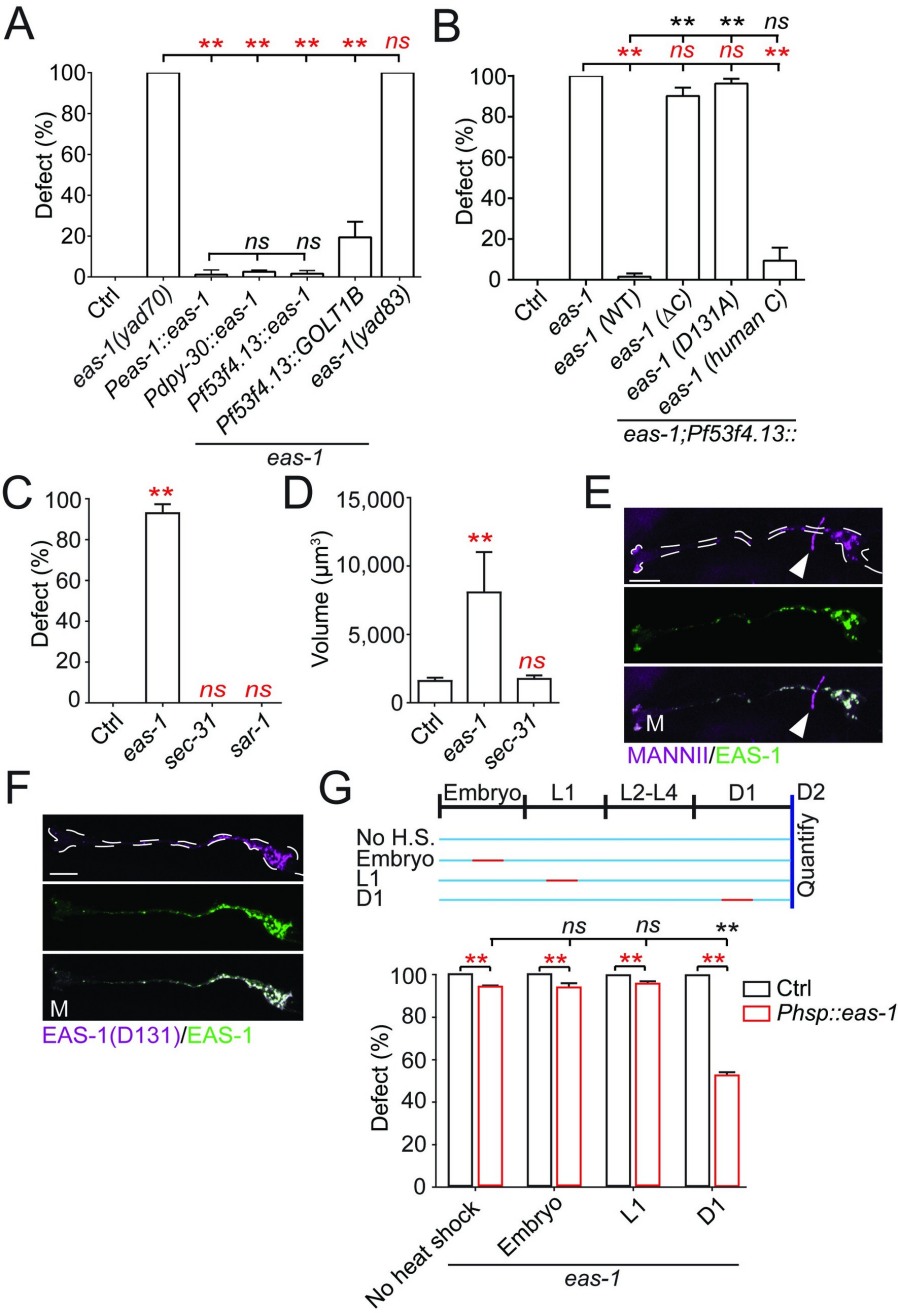
*eas-1* functions cell autonomously and in a conserved manner. (**A**) Rescue experiments of *eas-1* driven by its endogenous promoter (*Peas-1)*, a ubiquitously expressed promoter (*Pdpy-30*), and an AMsh-specific promoter (*Pf53f4*.*13*) in D2 adults. The genetic background is indicated in the figure and is WT unless otherwise stated. A human homolog of EAS-1, GOLT1B, was also tested. (**B**) Rescue experiments using different *eas-1* constructs—WT *eas-1*, *eas-1* lacking the carboxyl terminus (ΔC), *eas-1* with the D131A substitution on the carboxyl terminus (D131), and *eas-1* with a human carboxyl terminus (human C). Data were collected in D2 adult animals. (**C**) Percent enlarged AMsh and (**D**) volume of AMsh cell bodies after RNAi knockdown of *eas-1* and *sec-31*. All data were collected in D2 adult animals. (**E**) Confocal images of the *cis*-Golgi marker mRuby::MannII (top panel) and the fusion reporter GFP::EAS-1 (middle panel) expressed under the *Pf53f4*.*13* promoter in D1 adults. The bottom panel shows the merged image. The white arrows point to the AIY interneurons labeled by the co-injection marker *Pttx-3*::*RFP*. (**F**) Confocal images of the fusion reporter GFP::EAS-1(D131) (top panel) and the functional fusion reporter GFP::EAS-1 (middle panel) in D1 adults. The bottom panel shows the merged image. These reporters were expressed under the *Pf53f4*.*13* promoter. (**G**) Percentage of animals with enlarged AMsh cells after being heat-shocked for 3 hours at different stages of development shown in the schematic. Animals either expressed *eas-1* under a heat shock promoter (red) or nothing (black) in the *eas-1(yad70)* background. Scale bar, 10 μm. White dotted lines outline the AMsh cell. Data are represented as mean ± SD. One-way ANOVA, followed by Tukey HSD test, *p < 0.05, **p < 0.01. Each point represents 3 experiments of at least 50 worms. Underlying data for graphs can be found in S1 Data. AMsh, amphid sheath; ANOVA, analysis of variance; D1, day 1; D2, day 2; HSD, honestly significant difference; RNAi, RNA interference; SD, standard deviation; WT, wild-type.

There are 2 potential explanations for the enlarged AMsh phenotype in *eas-1(yad70)* mutants. It could be that regulation of glial growth is disrupted in the mutants; alternatively, AMsh cells may grow to fill up empty spaces left by surrounding cells that may die or respond to stress in other cells akin to reactive gliosis in other organisms [46]. To distinguish between these possibilities, we conducted rescue experiments in *eas-1(yad70)* mutants. It was found that expressing *eas-1* cDNA ectopically under its own promoter *Peas-1*, a ubiquitous promoter *Pdpy-30*, and an AMsh-specific promoter *Pf53f4*.*13* were all able to fully rescue the enlarged AMsh phenotype (Fig 2A). As *eas-1* can cell autonomously rescue the mutant phenotype, we conclude that AMsh cells are enlarged in *eas-1* mutants due to growth dysregulation rather than due to a response to surrounding cells. Furthermore, cell-specific expression of *eas-1* in AMsh cells also rescued the AWC-mediated chemotaxis toward benzaldehyde, AWA-mediated chemotaxis toward pyrazine, and ADL-mediated long-range repulsion from 1-octanol (S1F–S1H Fig), suggesting that the chemotaxis defects observed in *eas-1* mutants are primarily due to disrupted AMsh function, which, in turn, is required for proper function of these neurons [11]. While expression of the translational reporter *Peas-1*::*gfp*::*eas-1* using the same *eas-1* promoter as above showed broad expression throughout the worm (S3D Fig), AMsh and PHsh cells appear to be specifically affected when examined at the adult stage. Interestingly, ectopic expression of the human *eas-1* homolog (*GOLT1B)* under the AMsh-specific promoter *Pf53f4*.*13* was also able to rescue the enlarged AMsh phenotype in *eas-1(yad70)* animals, suggesting possible functional conservation (Fig 2A).

Next, to determine whether the carboxyl terminus that was affected in *yad70* was essential in the regulation of AMsh size, we conducted rescue experiments using various *eas-1* constructs with altered carboxyl termini. We found that AMsh-specific expression of *eas-1* with the last 13 amino acids deleted (ΔC) as well as *eas-1* with a highly conserved aspartate (D131) residue within these 13 amino acids replaced with an alanine (D131A) were unable to rescue the phenotype (Fig 2B; S3C and S3E Fig). We then generated an *eas-1* allele, *yad83*, with a D131A mutation and showed *eas-1*(*yad83)* displayed the same phenotype as that of *eas-1(yad70)* (Fig 2A). This suggests that the carboxyl terminus and the D131 within it are essential for *eas-1* function in regulating AMsh cell size. On the other hand, replacing the last 13 amino acids of *eas-1* with the human version (human C) was able to rescue the phenotype, further suggesting functional conservation of the *eas-1* carboxyl terminus (Fig 2B; S3C and S3E Fig).

*eas-1* is an essential gene, and deletion of the *eas-1* locus caused 100% embryonic lethality, which prevented further analysis of *eas-1* null allele phenotypes. To confirm that the enlarged AMsh cells we observed in *eas-1(yad70)* and *eas-1(yad83)* animals were due to a loss of function of the gene, we conducted RNAi knockdown in control animals. We found that although knockdown of *eas-1* caused strong lethality phenotypes, about 10% of animals were able to reach D2, and all of them recapitulated the phenotype observed in *yad70* mutants (Fig 2C and 2D; S3F Fig), suggesting that *yad70* represents a loss-of-function mutation.

Golgi transport 1B (GOLT1B) is a highly conserved membrane protein from yeast to human [13,14]. In studies conducted in yeast and rice, it was shown that its ortholog, Got1, likely functions in the early cisternae of the Golgi complex in addition to the ERES to facilitate anterograde ER–Golgi transport [13–15]. However, there are no published studies on this gene in animals, where the localization and function of GOLT1B are still unknown. To test whether the *eas-1* phenotypes were caused by a disruption of anterograde ER–Golgi transport given its reported role in yeast studies, we conducted RNAi knockdown of *sec-31* and *sar-1*, which are essential for anterograde ER–Golgi transport through COPII vesicles [47]. We observed strong lethality phenotypes (S3F Fig) in accordance with previous findings [48], but did not observe the enlarged AMsh phenotype found in *eas-1* knockdown animals (Fig 2C and 2D), suggesting that this phenotype is more specific to *eas-1*.

Use of the functional GFP::EAS-1 reporter revealed that it fully colocalized with the *cis*-Golgi marker mRuby::MannII [49] (Fig 2E; S3G Fig) and not the COPII vesicle marker SEC-24::mCherry [50] (S3H Fig), unlike what was previously reported for its homolog in yeast [14] and rice [15]. Furthermore, coexpression of the mutant construct mCherry::EAS-1(D131) with the functional GFP::EAS-1 reporter in AMsh cells showed almost complete colocalization as well (Fig 2F), suggesting that the mutations in the carboxyl terminus did not affect *eas-1* function by altering its localization.

To determine whether *eas-1* was transiently or continuously required for AMsh cell size regulation, we generated a *C*. *elegans* strain expressing *eas-1* under a heat shock promoter to temporally manipulate *eas-1* expression. We heat-shocked the worms at 33°C for 3 hours at the time periods indicated (Fig 2G) and found that induction of *eas-1* expression from day 1 (D1) was enough to significantly reduce the *eas-1(yad70)* phenotype penetrance compared to the no heat shock control when examined at D2. Furthermore, transient heat shocks during embryonic or L1 stages were unable to affect the phenotype when examined at D2, suggesting that continuous *eas-1* expression may be required to maintain proper AMsh cell size. Together, our results show that *eas-1* is required for preventing the overgrowth of AMsh cells.

### *rnf-145* functions downstream of *eas-1* by inhibiting *sbp-1* activation

**Fig 3:**
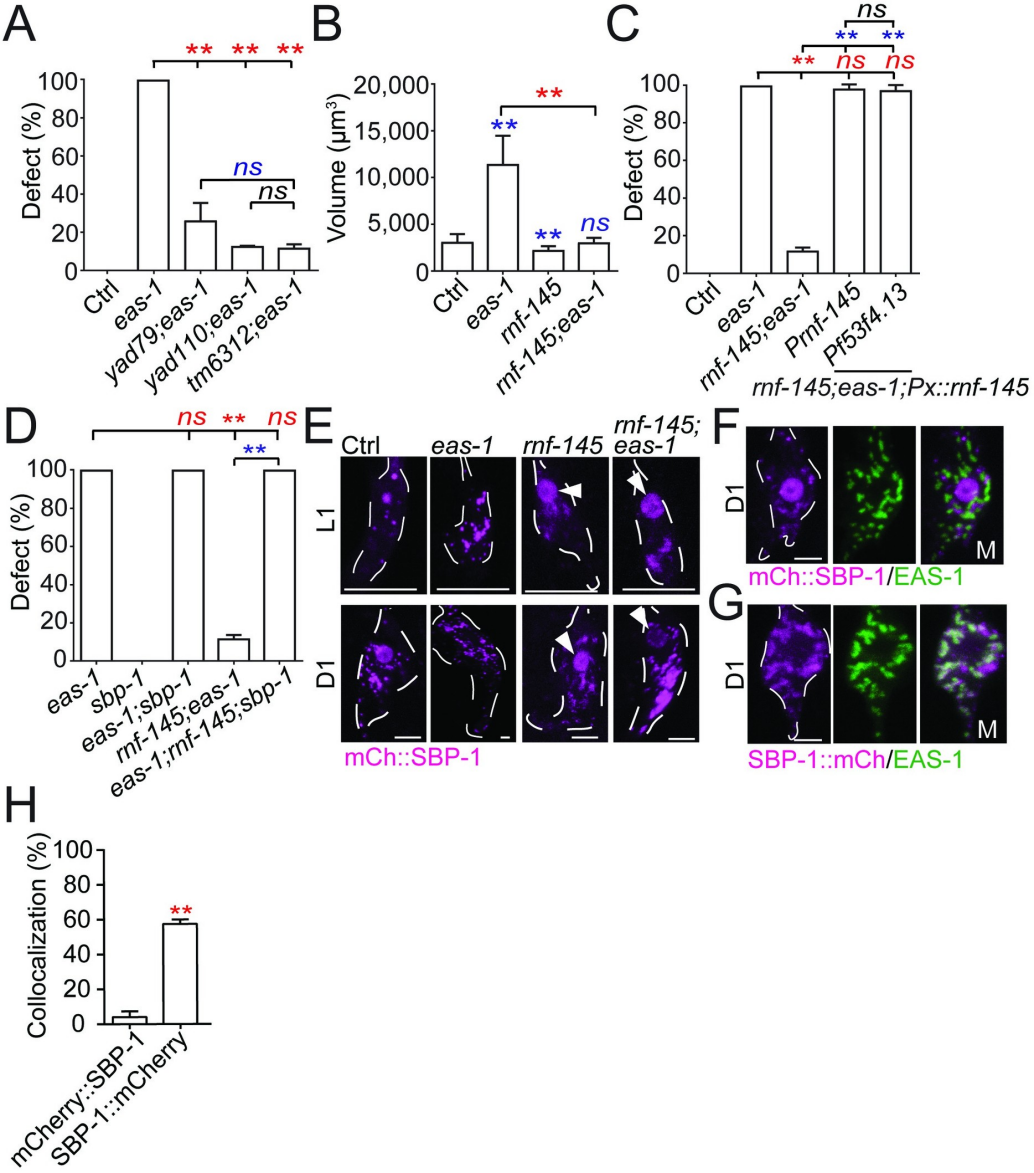
Components of the fatty acid homeostasis pathway lie downstream of *eas-1* in the regulation of cell size. (**A**) The percentage of enlarged AMsh cells in D2 animals, with the *eas-1(yad70)* allele and different *rnf-145* alleles. (**B**) The AMsh cell body volumes of D2 *eas-1(yad70)*, *rnf-145(yad110)*, or *eas-1(yad70)*;*rnf-145(yad110)* animals. (**C**) Rescue of the *eas-1(yad70);rnf-145(yad110)* mutant with expression of *rnf-145* under its endogenous promoter (*Prnf-145*) or the AMsh-specific promoter (*Pf53f4*.*13*). (**D**) The percentage of enlarged AMsh cells in D2 animals, with single, double, and triple mutant combinations of the *eas-1(yad70)* allele, *rnf-145(yad110)* allele, and *sbp-1(ep79)* allele. (**E**) Localization of SBP-1 in the AMsh cell using mCherry::SBP-1 in single and double mutant combinations of the *eas-1(yad70)* and *rnf-145(yad110)* alleles. Animals were imaged at the L1 and D1 adult stages. Arrows point to nuclear localization. (**F**) Coexpression of mCherry::SBP-1 and GFP::EAS-1 in the AMsh cells of WT D1 adults. (**G**) Coexpression of SBP-1::mCherry and GFP::EAS-1 in the AMsh cells of WT D1 adults. (**H**) Proportion of mCherry::SBP-1 or SBP-1::mCherry puncta that colocalize with GFP::RNF-145 puncta in the AMsh cells from the conditions in Fig 3F and 3G. Scale bar, 10 μm. White dotted lines outline the AMsh cell body. Data are represented as mean ± SD. *p < 0.05, **p < 0.01. **A**, **C**, and **D**: one-way ANOVA, followed by Tukey HSD test. Each point represents 3 experiments of at least 50 worms. **B**: one-way ANOVA, followed by Tukey HSD test. Each bar represents at least 10 animals. **H**: Student *t* test, *p < 0.05, **p < 0.01. Each bar represents at least 10 animals. Underlying data for graphs can be found in S1 Data. AMsh, amphid sheath; ANOVA, analysis of variance; D1, day 1; D2, day 2; HSD, honestly significant difference; SD, standard deviation; WT, wild-type.

To identify potential downstream components of *eas-1*, we conducted a suppressor screen using *eas-1(yad70)* animals and isolated 2 mutants *yad79* and *yad110* that both significantly suppressed the *eas-1(yad70)* phenotype in terms of penetrance and cell volume (Fig 3A and 3B). The *eas-1(yad70);rnf-145(yad110)* mutant also showed restored chemotaxis toward benzaldehyde and pyrazine in addition to avoidance of 1-octanol (S1F–S1H Fig). Both *yad79* and *yad110* caused nonsense mutations in an unnamed *C*. *elegans* gene, *Y119C1B*.*5*, which we named *rnf-145* based on its homology to the mammalian E3 ubiquitin ligase RNF145 (S4A and S4B Fig) [22,23]. A null allele of *rnf-145*(*tm6312)* with a deletion that causes a premature stop codon also exhibited similar suppression (Fig 3A; S4A and S4B Fig), supporting the conclusion that both *yad79* and *yad110* are null alleles of *rnf-145*, and loss of function of *rnf-145* can suppress *eas-1* phenotypes. Through rescue experiments, we found that expressing *rnf-145* under its own promoter as well as the AMsh-specific promoter *Pf53f4*.*13* in *eas-1(yad70);rnf-145(yad110)* double mutants could release the suppression of the enlarged AMsh phenotype (Fig 3C), suggesting that *rnf-145* is negatively regulated by *eas-1* in a cell-autonomous manner. *RNF145* was reported to be upstream of *SREBP-2* [22], so we wanted to determine if the *C*. *elegans* SREBP homolog *sbp-1* could be involved in AMsh cell size regulation. We generated an *eas-1(yad70);rnf-145(yad110);sbp-1(ep79)* triple mutant and found that loss of function of *sbp-1* was able to completely release the suppression of the *eas-1(yad70)* phenotype by *rnf-145(yad110)* (Fig 3D). *ep79* is a hypomorphic and temperature-sensitive *sbp-1* allele [51] that is viable at the permissive 20°C but lethal at 25°C, which may help explain the lack of phenotype in single mutants (Fig 3D; S4C Fig). *sbp-1(ep79)* animals that were cultured at 25°C after hatching were able to survive to adulthood, but did not exhibit a change in AMsh size when compared to the control animals (S4C Fig), which may again be due to the hypomorphic nature of the allele. However, the release of suppression by *sbp-1* in the triple mutant suggests that *eas-1* may regulate the size of AMsh cells though control of *sbp-1*/*SREBP* activation.

SREBPs are membrane-bound transcription factors that play essential roles in regulating lipid homeostasis, and cleavage of SREBPs release their N-terminal that could translocate to the nucleus and facilitate transcription [16,52]. The use of the translational fusion reporter *Psbp-1*::*gfp*::*sbp-1* shows that *sbp-1* is likely broadly expressed throughout the animal including many cells in the head (S4D Fig). Thus, we used nuclear localization as a marker to determine whether the activation of *sbp-1* is associated with AMsh growth. By expressing a functional mCherry::SBP-1 fusion reporter in AMsh cells, we found that in wild-type animals, SBP-1 does not accumulate in the nucleus during the early larval stages (L1 to L3) when the AMsh grow rapidly, but has strong nuclear localization when the animal enters the adult stage (Fig 3E; S4E and S4F Fig). Based on these observations, we hypothesize that the activation of SBP-1 may prevent the overgrowth of AMsh cells, and the regulation of AMsh cell size by *eas-1* and *rnf-145* may be through affecting SBP-1 nuclear localization. Indeed, we found that loss of function of *eas-1* abrogated the nuclear localization of SBP-1 in adult animals, and loss of function of *rnf-145* caused early-onset nuclear localization of SBP-1 in L1 animals (Fig 3E; S4F Fig). As expected, SBP-1 nuclear localization was restored in adult *eas-1(yad70)* animals when combined with the *rnf-145(yad110)* mutation (Fig 3E; S4F Fig). These results suggest that the regulation of SBP-1 activation by *eas-1* and *rnf-145* are important in the control of AMsh cell size.

In mammalian cells, SREBPs are initially located in the ER until activation, where they are shuttled to the *cis*-Golgi and cleaved to release the transcriptionally active bHLH domain [16,52,53]. Next, to determine if SBP-1 activation in *C*. *elegans* AMsh cells is like what was established in mammalian cells, we generated SBP-1 fusion reporters with mCherry tagged to the N-terminal and carboxyl terminus, respectively. We coexpressed them with the *cis*-Golgi-localized GFP::EAS-1 and found little colocalization with mCherry::SBP-1 (Fig 3F and 3H), while there was significantly stronger colocalization between GFP::EAS-1 and SBP-1::mCherry (Fig 3G and 3H). This suggests that the activation of SBP-1 occurs in the *cis*-Golgi as only the carboxyl terminus–tagged SBP-1, which labels the membrane-bound product after cleavage, shows *cis*-Golgi localization. Furthermore, no *trans*-Golgi localization of SBP-1::mCherry was detected (S4G Fig).

### *eas-1* mediates the translocation of RNF-145 from the cis- to trans-Golgi to activate SBP-1 during development

**Fig 4:**
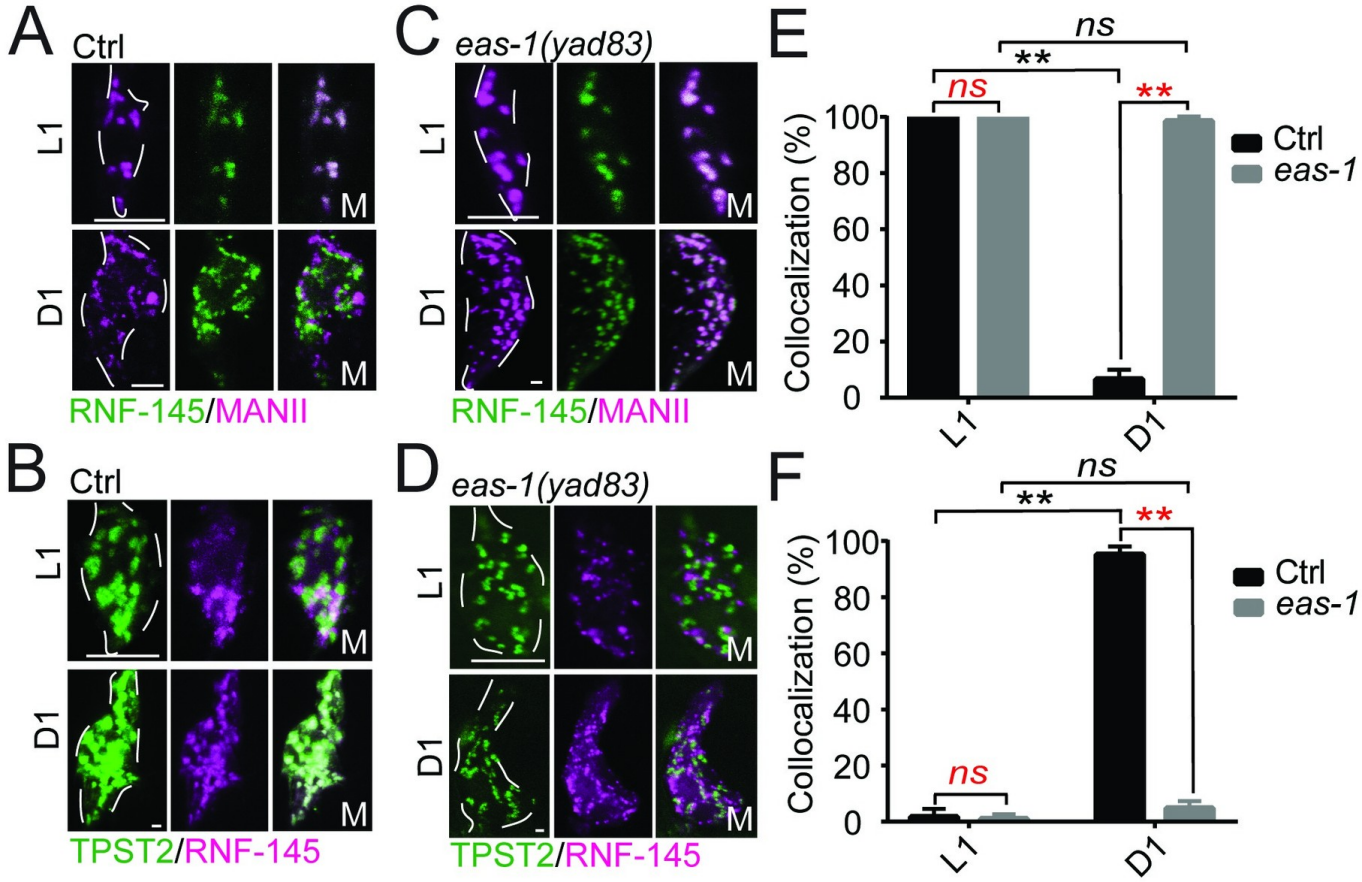
EAS-1 mediates the translocation of RNF-145 from the *cis*- to *trans*-Golgi. (**A**) Coexpression of GFP::RNF-145 with the *cis*-Golgi marker mRuby::MannII in the AMsh cells of WT animals. All animals in this figure were imaged at L1 and D1 adult stages. White dotted lines outline the AMsh cell body. (**B**) Coexpression of mCherry::RNF-145 with the *trans*-Golgi marker TPST-2::GFP in the AMsh cells of WT animals. (**C, D**) Expression of GFP::RNF-145 and mRuby::MannII (**C**) and mCherry::RNF-145 and TPST-2::GFP (**D**) in *eas-1(yad83)* animals. (**E**) Proportion of GFP::RNF-145 puncta that colocalize with mRuby::MannII puncta in the conditions from Fig **A** and **C**. (**F**) Proportion of mCherry::RNF-145 puncta that colocalize with TPST-2::GFP puncta in the conditions from Fig **B** and **D**. Scale bar, 10 μm. White dotted lines outline the AMsh cell body. One-way ANOVA, followed by Tukey HSD test. At least 10 animals were quantified for each condition. Underlying data for graphs can be found in S1 Data. AMsh, amphid sheath; ANOVA, analysis of variance; D1, day 1; HSD, honestly significant difference; WT, wild-type.

To better understand how *rnf-145* may regulate *sbp-1* activation, we generated a functional GFP::RNF-145 fusion reporter and coexpressed it with a *cis*-Golgi marker mRuby::MANII (Fig 4A; S5A Fig). RNF-145 showed strong localization in the *cis*-Golgi during the L1 stage, when the animal and its AMsh glia are rapidly growing, but appears to completely move out in D1 adults when growth starts to slow down (Fig 4A and 4E). To determine where RNF-145 translocates to in D1 animals, we coexpressed a functional mCherry::RNF-145 fusion reporter with a *trans*-Golgi marker TPST-2::GFP and found that almost all RNF-145 puncta have *trans*-Golgi localization in D1 adults, while few RNF-145 puncta were found in the *trans*-Golgi in L1 animals (Fig 4B and 4F; S5A Fig). Furthermore, RNF-145 colocalizes with EAS-1 in the *cis*-Golgi in L1 but not D1 adult animals (S5B and S5C Fig). As *rnf-145* is negatively regulated by *eas-1*, we further tested whether *eas-1* is involved in RNF-145 translocation during AMsh development. We found that loss of function of *eas-1* prevented the translocation of RNF-145 from *cis*- to *trans*-Golgi during development, and RNF-145 is localized to the *cis*-Golgi at all stages examined in the *eas-1(yad83)* background (Fig 4C–4F). As a control, we also coexpressed the mRuby::MannII and TPST-2::GFP markers in both control and *eas-1(yad70)* animals and found no strong colocalization in either genetic background, suggesting that the *cis*-Golgi and *trans*-Golgi compartments are distinct in *eas-1* mutants (S5D Fig). These data suggest that RNF-145 inhibits SBP-1 activation while in the *cis*-Golgi during early stages of development for faster AMsh growth, and growth slows when EAS-1 mediates the translocation of RNF-145 from the *cis*-Golgi to the *trans*-Golgi, thereby releasing inhibition on SBP-1 activation and slowing down growth.

**Fig 5:**
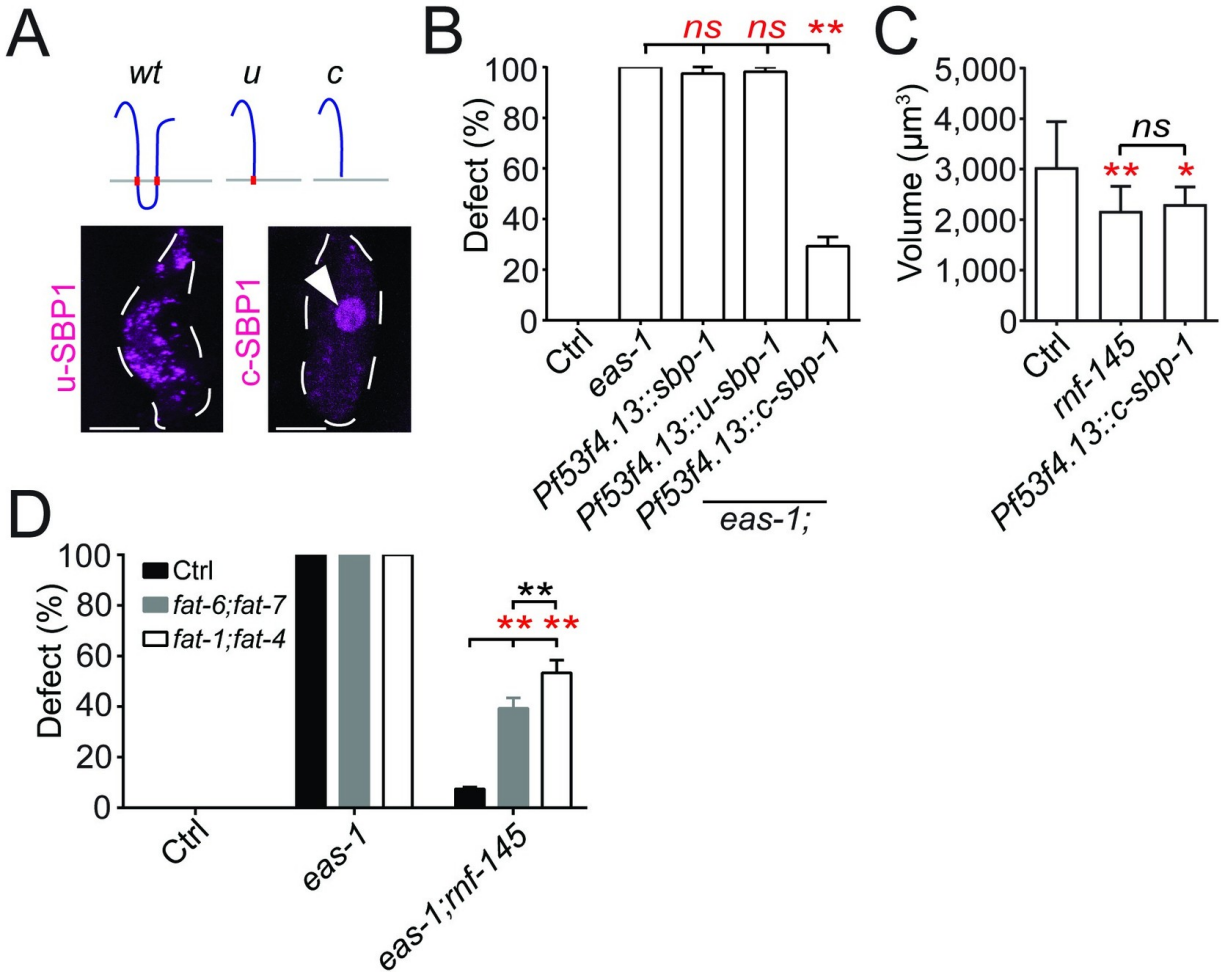
SBP-1 is involved in AMsh cell size regulation. (**A**) Schematic of the different *sbp-1* constructs used. On the bottom is the expression pattern of the *u* (uncleaved) and *c* (cleaved) *sbp-1* constructs in the AMsh cells of WT animals. White arrows point to nuclear localization. (**B**) Rescue of *eas-1(yad70)* animals with different *sbp-1* constructs detailed in Fig 5A. (**C**) The AMsh cell body volumes of D2 WT animals, *rnf-145(yad110)* animals and WT animals overexpressing *sbp-1 (c)* in AMsh cells. Each bar represents at least 10 animals. (**D**) Percentage of enlarged AMsh cells in WT, *eas-1(yad70)*, or *eas-1(yad70);rnf-145(yad110)* animals exposed to either control, *fat-1;fat4* or *fat-6;fat-7* RNAi knockdown conditions. Scale bar, 10 μm. White dotted lines outline the AMsh cell body. Data are represented as mean ± SD. *p < 0.05, **p < 0.01. Each point represents 3 experiments of at least 50 D2 adult worms unless otherwise stated. **B**: one-way ANOVA, followed by Tukey HSD test. **C**: Student *t* test. **D**: two-way ANOVA, followed by Tukey HSD test. Underlying data for graphs can be found in S1 Data. AMsh, amphid sheath; ANOVA, analysis of variance; D2, day 2; HSD, honestly significant difference; RNAi, RNA interference; SD, standard deviation; WT, wild-type.

Next, we wanted to test whether the activation of SBP-1 was important for and sufficient to reduce AMsh growth. We first expressed the nucleus-localized transcription-activating region of SBP-1 (c) [16] in the AMsh cells of *eas-1(yad70)* animals and found that it was able to suppress the enlarged AMsh phenotype (Fig 5A and 5B; S6A Fig). As controls, expression of full-length SBP-1 or the membrane-tethered transcription activating region of SBP-1 (u) in AMsh cells did not affect *eas-1(yad70)* phenotypes (Fig 5A and 5B; S6A Fig). We then tested whether driving SBP-1 activity in the nucleus was sufficient to reduce cell growth and found that expression of SBP-1 (c) in the AMsh cells of control animals could suppress their growth, and as a result, the size of AMsh cells in D2 animals was on average, about 25% smaller than those in control animals (Fig 5C). Similar small-sized AMsh cells were observed in *rnf-145* mutants, which have strong nuclear localization of SBP-1 in the L1 stage (Figs 3E and 5C). These results support the conclusion that SBP-1 activation is important to slow down AMsh growth, and the enlarged AMsh phenotypes in *eas-1* mutants likely arise in part due to a lack of SBP-1 activity.

#### The LC-PUFA eicosapentaenoic acid functions as a signal to prevent glial overgrowth

Previous studies have shown that SBP-1 activation is important for the expression of *fat-6* and *fat-7*, which are important Δ9 fatty acid desaturases that catalyze the production of stearic acid (18:0) into oleic acid (18:1) [54–56]. Thus, we tested whether these desaturases play a role in the regulation of AMsh cell size through RNAi knockdown experiments. We observed robust release of suppression in *eas-1(yad70);rnf-145(yad110)* animals when *fat-6* and *fat-7* expression are simultaneously knocked down (Fig 5D). Furthermore, this catalysis is important for the production of downstream LC-PUFAs, including the terminal product EPA (20:5) in *C*. *elegans* (S6B Fig) [55,56]. Thus, it is possible a downstream LC-PUFA product may be the downstream target of the *eas-1-rnf-145-sbp-1* pathway.

**Fig 6:**
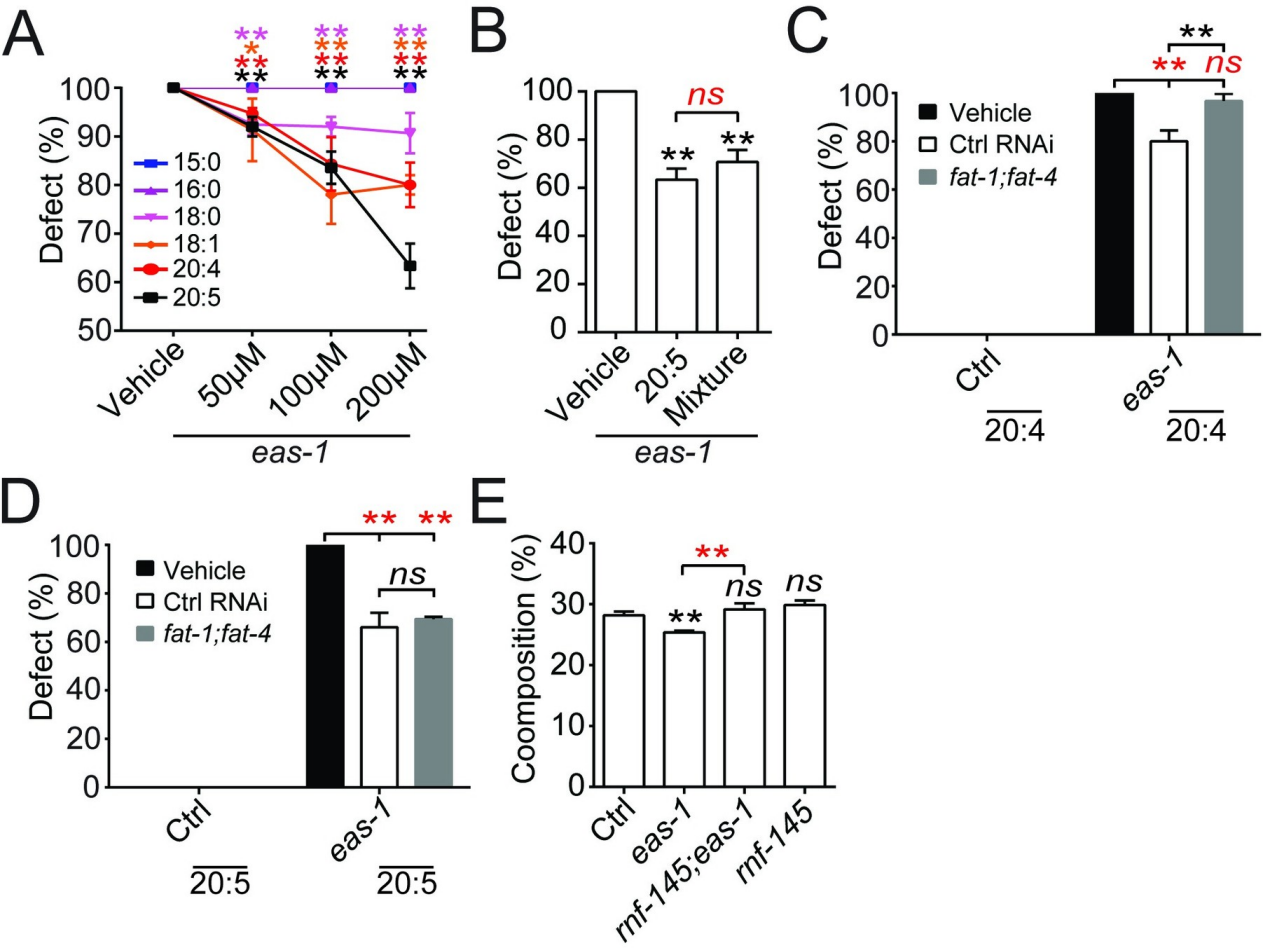
Polyunsaturated fatty acids act downstream of *eas-1* to inhibit AMsh cell growth. (**A**) Percentage of enlarged AMsh cells in *eas-1(yad70)* animals that were cultured on plates supplemented with 15:0, 16:0, 18:0, 18:1 (n-9), 20:4 (n-6), or 20:5 (n-3) at concentrations ranging from 0 μm to 200 μm. (**B**) Percentage of enlarged AMsh cells in *eas-1(yad70)* animals that were cultured on plates supplemented with the vehicle, 200 μm 20:5 (n-3), or a combination of 200 μm each of 15:0, 16:0, 18:0, 18:1 (n-9), 20:4 (n-6), and 20:5 (n-3). (**C, D**) Dietary supplementation of 20:4 (n-6) (**C**) and 20:5 (n-3) (**D**) in control and *eas-1(yad70)* animals that were exposed to *fat-1;fat-4* RNAi knockdown conditions. (**E**) Percent composition of 20:5 (n-3) in control, *eas-1(yad70)*, *rnf-145(yad110)*, or *eas-1(yad70);rnf-145(yad110)* animals obtained through LC/MS. Data were collected in mixed-stage whole animals. Each bar represents 3 biological replicates. Data are represented as mean ± SD. *p < 0.05, **p < 0.01. Each bar represents 3 experiments of at least 50 D2 adult worms unless otherwise stated. **A**, **C**, and **D**: two-way ANOVA, followed by Tukey HSD test. **B**: Student *t* test. **E**: one-way ANOVA, followed by Tukey HSD test. Underlying data for graphs can be found in S1 Data. AMsh, amphid sheath; ANOVA, analysis of variance; D2, day 2; HSD, honestly significant difference; LC/MS, liquid chromatography–mass spectrometry; RNAi, RNA interference; SD, standard deviation; WT, wild-type.

We next conducted dietary fatty acid supplementation experiments using several fatty acids in the pathway outlined in S6B Fig, including 15:0, 16:0, 18:0, 18:1 (n-9), 20:4 (n-6), and 20:5 (n-3). We found that supplementation of 18:0, 18:1, 20:4, and 20:5 showed suppression of *eas-1(yad70)* phenotypes in a dose-dependent manner, and 20:5 (n-3) has a significantly stronger suppression ability than any of the other fatty acids tested (Fig 6A; S6C Fig). As 20:5 is the terminal product of LC-PUFAs, we tested whether 18:0, 18:1, and 20:4 function by themselves or as materials for generating 20:5. We first examined the suppression ability of a mixture of all the fatty acids used (15:0, 16:0, 18:0, 18:1, 20:4, and 20:5) at the highest concentration of 200 μm each on *eas-1(yad70)* phenotypes. We found that it showed similar suppression ability as 200 μm of 20:5 alone (Fig 6B), suggesting that 20:5 may be a major downstream component in the regulation of AMsh size. On the other hand, dietary supplementation of any or all the fatty acids in wild-type animals did not reduce AMsh cell size (S6D Fig).

To further confirm whether 20:5 is the major product in this pathway regulating cell size, we tested the effects of knocking down the expression of the fatty acid desaturases *fat-1* and *fat-4*, which are required for the synthesis of 20:4 and 20:5 but not 18:0 or 18:1 (S6B Fig) [55,57]. We found that knockdown of *fat-1* and *fat-4* together showed significantly stronger release of suppression in *eas-1(yad70);rnf-145(yad110)* animals than both the RNAi control and *fat-6;fat-7* knockdown (Fig 5D), suggesting the importance of 20:4 or 20:5 in AMsh size regulation. To examine whether 20:4 and 20:5 both play a role in regulating AMsh size, we supplemented animals in *fat-1;fat-4* RNAi knockdown conditions with dietary 20:4 or 20:5, and we found that *fat-1* and *fat-4* expression abrogated the rescue of *eas-1(yad70)* animals by 20:4 (Fig 6C), while supplementation of 20:5 in *eas-1(yad70)* animals was able to rescue the phenotype of *fat-1;fat-4* knockdown animals to a similar degree as 20:5 supplementation animals with intact *fat-1* and *fat-4* (Fig 6D). These results support the conclusion that the terminal LC-PUFA 20:5 is the major fatty acid involved in AMsh size regulation. Finally, liquid chromatography–mass spectrometry (LC/MS) of bulk worms showed a significant reduction of 20:5 composition in *eas-1(yad70)* mutants, which was restored in the *eas-1(yad70);rnf-145(yad110)* double mutants (Fig 6E). Together, these data show that the LC-PUFA synthesis pathway lies downstream of *sbp-1*, where the fatty acid EPA appears to function as a potential signal for the inhibition of AMsh cell growth.

## Discussion

The regulation of cell size is a dynamic process throughout development that requires complex networks of positive and negative regulatory processes [1,2]. Through the study of *C*. *elegans* AMsh glial cells, we have uncovered a novel pathway of glial size regulation when AMsh glia reach their appropriate size and need to slow down growth, in which a conserved *cis*-Golgi membrane protein *eas-1/GOLT1B* functions as a key molecule in mediating the translocation of the E3 ligase RNF-145 from the *cis*-Golgi to the *trans*-Golgi, thereby removing the inhibition placed on *sbp-1*/*SREBP* in the *cis*-Golgi (S7 Fig). The activation of *sbp-1*/*SREBP* then promotes the synthesis of the LC-PUFA EPA to prevent glial overgrowth (S7 Fig). As the expression of the human *eas-1* homolog, *GOLT1B*, can rescue the enlarged AMsh phenotypes in *eas-1* mutants, the findings we reported here are likely to be an evolutionarily conserved mechanism of glial size regulation.

The regulation of SBP-1 activation by EAS-1 is through control of RNF-145 translocation from the *cis*- to *trans*-Golgi during development. The yeast EAS-1 homolog Got1 has been suggested to be involved in the regulation of COPII vesicles, which mediates anterograde transport from the *cis*- to *trans*-Golgi [13,14]. However, the function of *eas-1* in glial size regulation may not be through the general regulation of COPII vesicles, as RNAi knockdown of *sec-31* and *sar-1*, 2 key factors for the formation of COPII vesicles [58–60], did not affect the size of AMsh glia (Fig 2C).

As EAS-1 is highly enriched in the *cis*-Golgi membrane, it is possible that EAS-1 may be involved in the formation or budding of RNF-145-containing vesicles from the *cis*-Golgi, and those vesicles are likely to be COPII-independent vesicles.

Interestingly, unlike in *C*. *elegans*, RNF145 was localized to the ER when expressed in HeLa and HEK293T cells rather than the Golgi [22,23]. It was found that RNF145 ubiquitinates the ER membrane protein SCAP, an important regulator of SREBP activation, where it may inhibit its transport from the ER to the Golgi. Since SCAP mediates the transport of SREBPs from their site of synthesis in the ER to the Golgi where they are cleaved and activated [61], ubiquitination by RNF145 would likely inhibit SREBP activation. In *C*. *elegans*, it is unclear how RNF-145 functions to inhibit SBP-1 activation in the *cis*-Golgi and whether there may be crosstalk with the SCAP homolog SCP-1. In this case, RNF-145 may represent another layer of SBP-1 regulation coming from the Golgi after SBP-1 has been shuttled over.

Although EAS-1 is expressed in the *cis*-Golgi at all developmental stages, the EAS-1-dependent RNF-145 translocation only happens when animals reach the adult stage, which raises the question of what triggers the activation of EAS-1 to initiate the translocation. One of the best characterized pathways regulating cell growth in unicellular and multicellular organisms is the target of rapamycin (TOR) pathway, which has been found to play a role in most cell types tested [2, 62–64]. The TOR pathway can be activated by many extra- and intracellular signals during development, and it is possible that the TOR pathway may regulate the activation of EAS-1 in a developmental stage–dependent manner.

Furthermore, given the broad expression of *eas-1* and *sbp-1* (S3D and S4D Fig), it is unclear why the enlarged cell phenotypes appear to be specific to AMsh and PHsh cells. One possibility is that there are other intrinsic mechanisms in these cells that confer specificity to this mechanism of cell size regulation or make them uniquely susceptible to *eas-1* disruption. Another possibility is that this *eas-1*–*sbp-1* mechanism is more general, but individuals with severe phenotypes do not survive. Life span assays starting with L4 animals suggest that there is no noticeable difference in life span between wild-type and *eas-1(yad70)* animals (S8A Fig). On the other hand, when examining embryonic survival rates, only about 40% of embryos laid make it past the embryonic stage (S8B Fig). This suggests that the larval and adult animals observed may represent those with relatively less severe defects. Consistent with this, roughly 45% of *eas-1* embryos contained cells that were irregular in size and much larger than their wild-type counterparts (S8C and S8D Fig). Furthermore, all these irregular embryos were unable to survive while most of the wild-type-like *eas-1(yad70)* embryos did survive (S8E Fig). This suggests that perhaps the surviving adults had relatively weaker defects, especially since the enlarged AMsh cells are not necessary for survival of the animal; on the other hand, those with potentially more widespread cell size defects may not be able to survive past embryogenesis. The fact that only AMsh cells and not other nonessential cells exhibit the enlarged cell phenotype suggests that there may still be certain factors that make these glia particularly susceptible to *eas-1* disruption.

Another interesting finding of our study is the potential role of *sbp-1* in inhibiting cell growth. This appears counterintuitive, as the transcription is known to be a key activator of anabolic processes like cholesterol and fatty acid biosynthesis, and expression of a dominant positive version of SREBP-1a in mice leads to vastly enlarged hepatocytes that are distended by fat droplets [53,65]. Furthermore, it was reported that the silencing of SREBP led to reduced cell growth in both mammalian cell culture in vitro and drosophila cells in vivo [66]. However, it is known that LC-PUFAs like EPA can be precursors for signaling molecules like eicosanoids, where some of them have roles in regulating cell size [67,68]. Thus, it is possible that in certain cellular contexts, the *eas-1-rnf-145-sbp-1* pathway may promote the synthesis of certain lipids that have signaling roles to prevent cell overgrowth. Unlike *C*. *elegans*, mammals are unable to interconvert n-3 and n-6 PUFAs and need to acquire each from their diets. Given the importance of n-3 PUFAs during nervous system development, function, and disease, deficiencies have been linked to various neurodevelopmental, neurodegenerative, and cognitive disorders [69–72]. Thus, it is possible that there may be a glial aspect to nervous system disorders related to n-3 PUFA deficiencies, analogous to the role of the n-3 PUFA EPA in *C*. *elegans* glial development and function.

As human GOLT1B was able to rescue the *eas-1* phenotype cell autonomously, it is plausible that there may be functional conservation in mammals as well. EAS-1/GOLT1B may play a role in other cellular contexts involving RNF145 and SREBP2 like the liver [22–24], where it may play a more general role in regulating lipid homeostasis that is not limited to cell size regulation. The SREBP pathway was initially believed to be primarily regulated through negative feedback by cholesterol and oxysterols [73]. Recent studies have shown that the PI3K-Akt pathway is also involved in SREBP regulation [73], but the exact mechanisms regulating SREBP activation remain unclear. EAS-1/GOLT1B is broadly expressed, so it could represent another level of regulation of SREBP activation.

## Materials and methods

### Materials and data availability

Further information and requests for resources should be directed to and will be fulfilled by the lead contact, Dong Yan (dong.yan@duke.edu). *C*. *elegans* strains and plasmids generated in this study are available from the lead contact without restriction.

### *C*. *elegans* genetics

*C*. *elegans* strains were maintained on nematode growth media (NGM) plates using *Escherichia coli* OP50 as a food source. Animals were grown according to standard methods [74] at 20°C unless otherwise stated. Wild-type worms were of the Bristol N2 strain. All transgenes and strains are described in S1 Table. A complete list of DNA constructs are also included in S1 Table. *yadIs48* (*Pf53f4*.*13*::*GFP*) was used to visualize AMsh cells. *eas-1(yad70)* was the only mutant causing the significantly enlarged AMsh cell phenotype that was isolated from a visualized EMS mutagenesis screen of over 4,000 haploid genomes. The suppressor screen was conducted on over 2,000 haploid genomes by mutagenizing *eas-1(yad70)* animals using EMS and looking for any mutants that had wild-type AMsh cell size in older adults. Mutant alleles from both screens were sequenced using whole genome sequencing, and SNP-based mapping was conducted using the Hawaiian strain CB4856. Several *rnf-145* alleles were isolated, including *yad79* and *yad110*, and complementation analysis was performed to help identify *rnf-145* mutants. Mutations were finally confirmed through rescue experiments. All mutants isolated in this manuscript are recessive alleles.

### Cloning and constructs

All DNA expression constructs were generated using Gateway cloning technology (Invitrogen, Carlsbad, California, United States of America) and subsequently sequenced. *eas-1*, *rnf-145*, and *sbp-1* cDNA were amplified from a homemade genomic DNA pool or cDNA obtained from Dr. Yuji Kohara. A 2-kb promoter of *eas-1* and a 3-kb promoter of *rnf-145* were amplified from genomic DNA. In general, plasmid DNAs used in this study were injected at a concentration of 1 to 50 ng/μL with a *Pttx-3*::*RFP* co-injection marker injected at a concentration of 50 ng/μL.

### Microscopy

Representative images were acquired with a Zeiss LSM700 confocal microscope (Zeiss, Oberkochen, Germany) using a Plan-Apochromat 40×/1.4 objective. Worms were immobilized using 1.5% 1-phenoxy-2-propanol (TCI America, Portland, Oregon, USA) in M9 buffer and mounted on 5% agar slides. Three-dimensional reconstructions were done using Zeiss Zen software as maximum intensity projections. A Zeiss Axio Imager 2 microscope (Zeiss) equipped with Chroma HQ filters was used to score AMsh cell size defects. Each condition represented 3 experiments of at least 50 D2 animals each that were picked at random from the culture plate unless otherwise noted, in accordance with previous literature in *C*. *elegans*. AMsh cell volume was quantified by measurement of the 3D reconstructions using Imaris (Bitplane, Belfast, United Kingdom). We defined the cell body for the purpose of quantification to be the area enclosing the nucleus to the part just before it tapers into a process of relatively constant width. As the mutant phenotypes are binary, where cells are either wild-type sized or 3 to 4 times larger than their wild-type counterpart, we scored the penetrance percentages by eye for more efficient quantification. Colocalization of fluorescent puncta was conducted manually using at least 5 animals, where results are given as the percentage of overlapping puncta out of the total number of the stated puncta. For the mCherry::SBP-1 nuclear signal quantification, the nuclear to nonnuclear ratio was measured using Fiji [75].

### Transmission electron microscopy

For transmission electron microscopy (TEM), samples were fixed quickly for 24 hours in 2% paraformaldehyde (Sigma-Aldrich, St. Louis, Missouri, USA), 2.5% glutaraldehyde (Ted Pella, Redding, California, USA), and 0.003% CaCl2 (Sigma-Aldrich) in sodium cacodylate buffer (Sigma-Aldrich), post-fixed in 2% osmium tetroxide (Ted Pella), dehydrated in an acetone dilution series (30%, 50%, 70%, and 90%, followed by 3 × 100%), and embedded with Eponate 12 resin (Electron Microscopy Sciences, Hatfield, Pennsylvania, USA). Sections for TEM were cut to a thickness of 70 nm with diamond knives and a Leica EM UC7 ultramicrotome (Leica, Wetzlar, Germany). The sections were stained with either 2% (w/v) aqueous uranyl acetate and 1% (w/v) lead citrate. Specimens were viewed with a TecnaiG2 Spirit 120kV (Thermo Fisher, Waltham, Massachusetts, USA) transmission electron microscope.

Due to size variability between wild-type and *eas-1(lf)* animals (S1B Fig), the relative position of the AMsh cell body on the animal was used to determine where to section the animals. The relative position of the AMsh cell body was measured to be consistent and similar between both genotypes, at around 0.12 times the total length of the animal from the nose tip (S1J Fig). Sections around this region were used for TEM imaging, and the AMsh cell bodies were identified in reference to micrographs from WormAtlas [76].

### Dye filling of amphid neurons

D1 worms were washed and incubated on a slow rotator with DiI (Molecular Probes, Eugene, Oregon, USA) at a concentration of 10 μg/mL for 2 hours at room temperature. After 3 washings, animals were mounted and visualized on the Zeiss LSM700 confocal microscope using the LP560 filter. Of the 12 amphid neurons in each sensilla, the ADL, ASH, ASI, ASJ, ASK, and AWB neurons are labeled and counted.

### Chemotaxis assays

Chemotaxis assays were conducted on circular plates as previously described [26,77]. To test for neuron function, we used ethanol as a diluent with 0.5% benzaldehyde and 1% pyrazine (Sigma-Aldrich) as odor attractants, as well as 100% 1-octanol as a repulsive odor. All experiments were done using at least 100 D2 adult worms each. Chemotaxis index was calculated by

$$\frac{worms\;in\;attractant\;region-worms\;in\;control\;region}{total\;animals}$$

### RNAi experiments

RNAi knockdown was done by feeding *E*. *coli* strain HT115 (DE3) from the Ahringer library (Source BioScience, Nottingham, UK) expressing double-stranded RNA fragments homologous to the target gene. The RNAi-expressing bacteria were cultured overnight at 37°C in LB with ampicillin (100 μg/mL). Subsequently, 30 μL of the overnight culture was added to 3-mL LB with ampicillin and incubated for 2 hours at 37°C before 1-μL 1M IPTG was added. The culture was then incubated for 4 hours before being mixed with 10-μL ampicillin (100 mg/mL) and 30 μL 1M IPTG and plated onto NGM plates. Different RNAi cultures were also mixed at this stage for double knockdown experiments. The plates were incubated overnight at 37°C. Gravid adults were bleached and their embryos were added to the RNAi plates. Animals would feed for at least 2 generations before they were quantified. GFP RNAi and the empty L4440 vector were used as positive and negative controls, respectively, for every experiment.

### Fixed staining of animals

D2 animals were fixed using 0.5% PFA (Sigma-Aldrich) for 5 minutes and freeze-thawed twice. CellTrace BODIPY TR methyl ester dye (Thermo Fisher) was added at a final concentration of 100 μm and incubated overnight at 4°C. Nile Red (Sigma-Aldrich) was added at a final concentration of 1 μg/mL and incubated for 1 hour at room temperature. After 3 washings, animals were mounted and visualized on the Zeiss LSM700 confocal microscope using the LP560 filter.

### Heat shock experiments

Heat shock experiments were carried out in animals expressing *Phsp*::*eas-1* or controls without it and were heat-shocked at 33°C for 3 hours at the stage specified (Fig 2G). All animals were quantified during the D2 adult stage.

### Dietary fatty acid supplementation

Feeding experiments were carried out in the protocol outlined by Deline and colleagues [78]. Fatty acids were supplemented in *C*. *elegans* NGM at concentrations ranging from 50 μm to 200 μm and were subsequently seeded with OP50 or RNAi-expressing bacteria. Animals were grown for at least 2 generations on the plates before quantification. All plates used were made fresh the day before. All fatty acids used were obtained from Thermo Fisher.

### Fatty acid analysis by LC/MS

For fatty acid analysis of *C*. *elegans*, lipid extraction and alkaline hydrolysis were performed according to a previously published protocol with modifications [79]. Briefly, each *C*. *elegans* pellet (approximately 50 mg) was suspended in a 3.8-mL mixture of CHCl3/MeOH/0.5 N NaOH (1:2:0.8, v/v/v) and heated at 60°C for 1 hour. After centrifugation at 3,000×g for 10 minutes, the supernatant was transferred to a fresh glass tube and was converted to a 2-phase Bligh/Dyer mixture consisting of chloroform/methanol/water (2:2:1.8, v/v/v) by adding appropriate volumes of chloroform and water. After centrifugation at 3,000×g for 10 minutes, the lower phase was collected and dried under nitrogen gas. The dried lipid extracts were stored at −20°C until LC/MS analysis.

Normal phase LC-electrospray ionization (ESI)/MS of the lipid extracts was performed using an Agilent 1200 Quaternary LC system coupled to a high-resolution TripleTOF5600 mass spectrometer (Sciex, Framingham, Massachusetts, USA). Chromatographic separation was performed on an Ascentis Silica HPLC column, 5 μm, 25 cm × 2.1 mm (Sigma-Aldrich). Elution was achieved with mobile phase A, consisting of chloroform/methanol/aqueous ammonium hydroxide (800:195:5, v/v/v), mobile phase B, consisting of chloroform/methanol/water/aqueous ammonium hydroxide (600:340:50:5, v/v/v/v), and mobile phase C, consisting of chloroform/methanol/water/aqueous ammonium hydroxide (450:450:95:5, v/v/v/v), over a 40-minute long run, performed as follows: 100% mobile phase A was held isocratically for 2 minutes and then linearly increased to 100% mobile phase B over 14 minutes and held at 100% B for 11 minutes. The mobile phase composition was then changed to 100% mobile phase C over 3 minutes and held at 100% C for 3 minutes and finally returned to 100% A over 0.5 minutes and held at 100% A for 5 minutes. The LC eluent (with a total flow rate of 300 μL/min) was introduced into the ESI source of the high-resolution TF5600 mass spectrometer. MS and tandem mass spectrometry (MS/MS) were performed in negative ion mode, with the full-scan spectra being collected in the m/z 200 to 2,000 range. The MS settings are as follows: ion spray voltage (IS) = −4,500 V (negative ion mode), curtain gas (CUR) = 20 psi, ion source gas 1 (GS1) = 20 psi, de-clustering potential (DP) = −55 V, and focusing potential (FP) = −150 V. Nitrogen was used as the collision gas for MS/MS experiments. Data analysis was performed using Analyst TF1.5 software (Sciex). The peak areas of the extracted ion chromatograms of the [M-H]− ions of each free fatty acids are used for relative abundance comparison.

### Life span assay

L4 animals were cultured at 20°C on NGM plates with added FUDR. At least 50 animals were used for each experiment, and the number of surviving animals was counted daily until none were left. Three independent replicated were done for each sample.

### Embryonic survival assay

Gravid adults were added to deposit embryos on a fresh NGM plate and were removed after 3 hours. The embryos were scored and incubated for 2 days at 20°C, and any remaining unhatched embryos were scored as dead.

For correlation of cell size distribution to survival, early *eas-1(yad70)* embryos were labeled by the P*mex-5*::*mCherry-C1*::*PLC(delta)-PH*::*tbb-2 3'UTR* reporter [80], which labels all cell membranes in the embryo. The embryos were sorted into seeded NGM plates based on whether they have the irregular cell size phenotype, and the number of surviving animals for each condition was quantified 2 days later. Triplicates of at least 50 embryos each were conducted.

### Statistical analysis

Data were analyzed using 2-tailed Student *t* test, 1-sample *t* test, and 1-way or 2-way ANOVA followed by Tukey honestly significant difference (HSD) test in GraphPad Prism 7 (GraphPad Software, La Jolla, California, USA). Raw data used in the graphs are contained in S1 Data.

## Supporting information

S1 Fig Characterization of *eas-1(yad70)* mutants. (**A**) The ratio of *eas-1(yad70)* to WT AMsh cell body volume at each respective stage measured. (**B**) The total length of D2 WT and *eas-1(yad70)* animals. Each bar represents at least 10 worms. (**C**) The percentage of D2 WT or *eas-1(yad70)* animals with enlarged AMsh cells after food deprivation since L4. Data sets comprised of control unstarved worms are filled white, while those comprised of starved filled are colored black. Each bar represents 3 experiments of 50 worms each. Two-way ANOVA, followed by Tukey HSD test, *p < 0.05, **p < 0.01. (**D**) DiI staining of amphid neurons in WT or *eas-1(yad70)* D1 adult animals. Arrowheads point to each visible amphid neuron cell body. As these 12 neurons are close to each other, in the projection images, 2 neurons are difficult to present, but we clearly observed 12 neurons under the microscope. Scale bar, 10 μm. White dotted lines outline the worm. (**E**) Number of amphid neurons stained by DiI in WT or *eas-1(yad70)* D1 adult animals. One-sample *t* test, *p < 0.05, **p < 0.01. (**F–H**) Chemotaxis indexes of WT, *eas-1(yad70)*, *rnf-145(yad110);eas-1(yad70)*, and *eas-1(yad70);Pf53f4*.*13*::*eas-1* animals in response to 0.5% benzaldehyde (**F**), 1% pyrazine (**G**), or 100% 1-octanol (**H**). Each bar represents 3 experiments of at least 100 animals. One-way ANOVA, followed by Tukey HSD test, *p < 0.05, **p < 0.01. Underlying data for graphs can be found in S1 Data. AMsh, amphid sheath; ANOVA, analysis of variance; D2, day 2; HSD, honestly significant difference; WT, wild-type.
(TIF)

S2 Fig Enlarged AMsh of *eas-1(yad70)* mutants do not appear to be due to accumulation of large vacuoles, lysosomes, LROs, or lipid droplets. (**A**) Expression of the lysosomal membrane fusion protein LMP-1::GFP in the AMsh of D1 adult control and *eas-1(yad83)* animals. Scale bar, 10 μm. (**B**) Expression of the fusion reporter mCherry::GLO-1 in the AMsh of D1 adult control and *eas-1(yad83)* animals. (**C**) Percentage of D2 animals with enlarged AMsh cells after RNAi knockdown of control, *glo-1* or *glo-3* in either WT or *eas-1(yad70)* backgrounds. Each point represents 3 experiments of at least 50 animals. (**D, E**) Staining of PFA-fixed D2 adult animals with (**D**) CellTrace BODIPY TR or (**E**) Nile Red in control and *eas-1(yad70)* backgrounds. White arrowheads point to dye puncta within the AMsh cell. Left 3 panels are from a single focus plane: M, merged; 3D, 3D reconstruction. Scale bar, 10 μm. (**F**) Schematic of where TEM cross-sections were cut to show the AMsh cell bodies. TL, the measured total length from the nose to tail. Sections were cut in the region around 0.12 total lengths from the nose tip and imaged by TEM. Scale bar, 10 μm. White dotted lines outline the AMsh cell body. Data are represented as mean ± SD. Underlying data for graphs can be found in S1 Data. AMsh, amphid sheath; D1, day 1; D2, day 2; LRO, lysosome-related organelle; RNAi, RNA interference; SD, standard deviation; TEM, transmission electron microscopy; WT, wild-type.
(TIF)

S3 Fig Loss of function in *eas-1* causes enlarged AMsh glia. (**A**) RT-PCR products of *eas-1* in WT and *eas-1(yad70)* mutants. *ama-1* was used as a housekeeping gene, and the right column contains PCR fragments amplified from WT genomic DNA. (**B**) Genomic organization of *eas-1*. The point mutations in the *yad70* and *yad83* alleles are colored red. Part of the WT and *yad70* exons after the point mutation site are highlighted. (**C**) The amino acid sequences of *eas-1* and its homologs in *Saccharomyces cerevisiae*, *Mus musculus*, and *Homo sapiens*. The *yad70* and *yad83* alleles are also included. The conserved D131 residue is colored green, while differing residues in the alleles are colored red. (**D**) Expression of the *Peas-1*::*GFP*::*eas-1* translational reporter in L1 (top panel) and D1 animals (bottom panel). Dashed lines outline the shape of the worm. Scale bar, 10 μm. (**E**) Schematic of different *eas-1* constructs from Fig 2B—WT *eas-1*, *eas-1* lacking the carboxyl terminus (ΔC), *eas-1* with the D131A substitution (D131A), and *eas-1* with a human carboxyl terminus (human C). (**F**) Percentage survival of WT animals after RNAi knockdown of control, *eas-1*, and *sec-31*. Embryos were plated and the percentage of worms that successfully hatched were quantified. (**G**) Rescue experiments using either *Pf53f4*.*13*::*eas-1* or *Pf53f4*.*13*::*GFP*::*eas-1* in D2. Genetic background is WT unless otherwise indicated. (**H**) Confocal images of the fusion reporter GFP::EAS-1 and the COPII vesicle marker SEC-24::mCherry in the AMsh of D1 adults. Scale bar, 10 μm for top figures, 2 μm for zoomed in figures below. White dotted lines outline the AMsh cell body. Data are represented as mean ± SD. One-way ANOVA, followed by Tukey HSD test, *p < 0.05, **p < 0.01. Each point represents 3 experiments of at least 50 animals. Underlying data for graphs can be found in S1 Data. AMsh, amphid sheath; ANOVA, analysis of variance; D1, day 1; D2, day 2; HSD, honestly significant difference; RNAi, RNA interference; RT-PCR, reverse transcription PCR; SD, standard deviation; WT, wild-type.
(TIF)

S4 Fig *rnf-145* alleles and localization of SBP-1. (**A**) Genomic organization of *rnf-145* on top, with the point mutations of the *yad79* and *yad110* alleles labeled in red and the span of deletion of the *tm6312* allele denoted with a black line. (**B**) The predicted amino acid structure of *rnf-145* and its homologs in *Mus musculus*, *Homo sapiens*, and *Drosophila melanogaster*. More highly conserved regions are labeled in red. (**C**) The volumes of AMsh cell bodies in WT and *sbp-1(ep79)* animals in μm^3^. *sbp-1(ep79)* mutants are temperature sensitive, and after hatching were cultured at the permissive 20°C and the nonpermissive 25°C, respectively, before imaging. Each point represents at least 10 animals. (**D**) Expression of the *Psbp-1*::*GFP*::*sbp-1* translational reporter in L1 (top panel) and D1 animals (bottom panel). Dashed lines outline the shape of the worm. (**E**) Coexpression of mCherry::SBP-1 and the nuclear marker H2B::GFP in the AMsh cells of WT D1 adults. White dotted lines outline the AMsh cell body. (**F**) Quantification of the percentage total signal of mCherry::SBP-1 signal that colocalizes with H2B::GFP. Each bar represents quantification of 6 worms. (**G**) Coexpression of SBP-1::mCherry and the *trans*-Golgi marker GFP::TSPT-2 in the AMsh cells of WT D1 adults. White dotted lines outline the AMsh cell body. Scale bar, 10 μm. Data are represented as mean ± SD. Student t test, *p < 0.05, **p < 0.01. Underlying data for graphs can be found in S1 Data. AMsh, amphid sheath; D1, day 1; SD, standard deviation; WT, wild-type.
(TIF)

S5 Fig EAS-1 colocalizes with RNF-145 in L1 animals. (**A**) Rescue of *rnf-145(yad110);eas-1(yad70)* using the fusion reporters *Pf53f4*.*13*::*GFP*::*rnf-145* and *Pf53f4*.*13*::*mCherry*::*rnf-145*. Data are represented as mean ± SD. One-way ANOVA, followed by Tukey HSD test, *p < 0.05, **p < 0.01. Each point represents 3 experiments of at least 50 animals. (**B**) Coexpression of GFP::EAS-1 with mCherry::RNF-145 in the AMsh cells of WT animals. Animals were imaged at L1 and D1 adult stages. Scale bar, 10 μm. (**C**) Proportion of mCherry::RNF-145 puncta that colocalize with GFP::EAS-1 puncta in during the L1 and D1 stages. Data are represented as mean ± SD. Student *t* test, *p < 0.05, **p < 0.01. At least 10 animals were quantified for each condition. (**D**) Coexpression of TPST-2::GFP and mRuby::MannII in AMsh cells of control and *eas-1(yad70)* D1 adult animals. Scale bar, 10 μm. White dotted lines outline the AMsh cell body. Underlying data for graphs can be found in S1 Data. AMsh, amphid sheath; ANOVA, analysis of variance; D1, day 1; HSD, honestly significant difference; SD, standard deviation; WT, wild-type.
(TIF)

S6 Fig SBP-1 activation regulates LC-PUFA biosynthesis. (**A**) Percentage of animals with enlarged AMsh cells after ectopic expression of the different *sbp-1* constructs in the AMsh cells of D2 WT animals. Each point represents 3 experiments of at least 50 animals. (**B**) Schematic of the LC-PUFA synthesis pathway in *C*. *elegans*. (**C**) The AMsh cell body volumes of D2 control, *eas-1(yad70)* and *eas-1(yad70)* supplemented with 200 μM EPA. Fifteen D2 animals were randomly picked quantified for each condition. (**D**) The AMsh cell body volumes of D2 WT animals supplemented with dietary polyunsaturated fatty acids at a concentration of 200 μm each. Mix represents a mixture of all the above fatty acids at a concentration of 200 μm each. Each bar represents at least 10 animals. Data are represented as mean ± SD. One-way ANOVA, followed by Tukey HSD test, *p < 0.05, **p < 0.01. Underlying data for graphs can be found in S1 Data. AMsh, amphid sheath; ANOVA, analysis of variance; D2, day 2; EPA, eicosapentaenoic acid; HSD, honestly significant difference; LC-PUFA, long-chain polyunsaturated fatty acid; SD, standard deviation; WT, wild-type.
(TIF)

S7 Fig Model of *eas-1* regulation of cell size. Model of how *eas-1/GOLT1B* may regulate growth through release of inhibition on *sbp-1/SREBP*.
(TIF)

S8 Fig *eas-1* mutants exhibit embryonic lethality. (**A**) Percentage of control (black) or *eas-1(yad70)* (magenta) animals surviving at each time point. Three replicates of at least 50 worms were quantified for each time point. (**B**) Percentage of control and *eas-1(yad70)* animals that survive past the embryonic stage. Each bar represents 3 experiments of at least 60 embryos. (**C**) *eas-1(yad70)* embryos that exhibit WT-like (shown top) and irregular (bottom) cell sizes at 20°C. Labeled by the P*mex-5*::*mCherry-C1*::*PLC(delta)-PH*::*tbb-2 3'UTR* reporter that labels all cell membranes in the embryo. Scale bar, 10 μm. (**D**) Percentage of *eas-1(yad70)* embryos with irregular cell sizes. (**E**) Percentage survival of *eas-1(yad70)* animals past embryogenesis based on whether they start with irregular cell sizes. Each bar represents 3 experiments of at least 50 embryos. Data are represented as mean ± SD. Student *t* test, *p < 0.05, **p < 0.01. Underlying data for graphs can be found in S1 Data. SD, standard deviation; WT, wild-type.
(TIF)

S1 Table Strain list. Table of strains and plasmids used in this study.
(DOCX)

S1 Data Raw data used for all figures in this study. (XLSX)

## Abbreviations

AMsh: amphid sheath
bHLH: basic helix-loop-helix
CUR: curtain gas
D1: day 1
D2: day 2
D3: day 3
DP: de-clustering potential
EPA: eicosapentaenoic acid
ERES: ER exit site
ESI: electrospray ionization
FP: focusing potential
GOLT1B: Golgi transport 1B
GS1: ion source gas 1
HSD: honestly significant difference
IS: ion spray voltage
LC/MS: liquid chromatography–mass spectrometry
LC-PUFA: long-chain polyunsaturated fatty acid
LRO: lysosome-related organelle
LXR: liver X receptor
MS/MS: tandem mass spectrometry
NGM: nematode growth media
PHsh: phasmid sheath glia
RNAi: RNA interference
RNF145: RING finger protein 145
RT-PCR: reverse transcription PCR
SREBP: sterol regulatory element-binding protein
TEM: transmission electron microscopy
TOR: target of rapamycin

## References

[1] Echave P, Conlon IJ, Lloyd AC. Cell size regulation in mammalian cells. Cell Cycle. 2007;6(2):218–24. 10.4161/cc.6.2.3744 . 17245129

[2] Lloyd AC. The regulation of cell size. Cell. 2013;154(6):1194–205. 10.1016/j.cell.2013.08.053 . 24034244

[3] Schmoller KM, Skotheim JM. The Biosynthetic Basis of Cell Size Control. Trends Cell Biol. 2015;25(12):793–802. 10.1016/j.tcb.2015.10.006 . 26573465 PMC6773270

[4] Nurse P. Genetic control of cell size at cell division in yeast. Nature. 1975;256(5518):547–51. 10.1038/256547a0 . 1165770

[5] Johnston GC, Pringle JR, Hartwell LH. Coordination of growth with cell division in the yeast Saccharomyces cerevisiae. Exp Cell Res. 1977;105(1):79–98. 10.1016/0014-4827(77)90154-9 . 320023

[6] Sherman DL, Brophy PJ. Mechanisms of axon ensheathment and myelin growth. Nat Rev Neurosci. 2005;6(9):683–90. 10.1038/nrn1743 . 16136172

[7] Barres BA. The mystery and magic of glia: a perspective on their roles in health and disease. Neuron. 2008;60(3):430–40. 10.1016/j.neuron.2008.10.013 . 18995817

[8] Hanani M. Satellite glial cells in sensory ganglia: from form to function. Brain Res Brain Res Rev. 2005;48(3):457–76. 10.1016/j.brainresrev.2004.09.001 . 15914252

[9] Ward S, Thomson N, White JG, Brenner S. Electron microscopical reconstruction of the anterior sensory anatomy of the nematode Caenorhabditis elegans.?2UU. J Comp Neurol. 1975;160(3):313–37. 10.1002/cne.901600305 . 1112927

[10] Perkins LA, Hedgecock EM, Thomson JN, Culotti JG. Mutant sensory cilia in the nematode Caenorhabditis elegans. Dev Biol. 1986;117(2):456–87. 10.1016/0012-1606(86)90314-3 . 2428682

[11] Bacaj T, Tevlin M, Lu Y, Shaham S. Glia are essential for sensory organ function in C. elegans. Science. 2008;322(5902):744–7. 10.1126/science.1163074 18974354 PMC2735448

[12] Oikonomou G, Perens EA, Lu Y, Watanabe S, Jorgensen EM, Shaham S. Opposing activities of LIT-1/NLK and DAF-6/patched-related direct sensory compartment morphogenesis in C. elegans. PLoS Biol. 2011;9(8):e1001121 10.1371/journal.pbio.1001121 21857800 PMC3153439

[13] Conchon S, Cao X, Barlowe C, Pelham HR. Got1p and Sft2p: membrane proteins involved in traffic to the Golgi complex. EMBO J. 1999;18(14):3934–46. 10.1093/emboj/18.14.3934 10406798 PMC1171469

[14] Lorente-Rodriguez A, Heidtman M, Barlowe C. Multicopy suppressor analysis of thermosensitive YIP1 alleles implicates GOT1 in transport from the ER. J Cell Sci. 2009;122(Pt 10):1540–50. 10.1242/jcs.042457 19383723 PMC2680100

[15] Wang Y, Liu F, Ren Y, Wang Y, Liu X, Long W, et al GOLGI TRANSPORT 1B Regulates Protein Export from the Endoplasmic Reticulum in Rice Endosperm Cells. Plant Cell. 2016;28(11):2850–65. 10.1105/tpc.16.00717 27803308 PMC5155349

[16] Horton JD, Goldstein JL, Brown MS. SREBPs: activators of the complete program of cholesterol and fatty acid synthesis in the liver. J Clin Invest. 2002;109(9):1125–31. 10.1172/JCI15593 11994399 PMC150968

[17] Eberle D, Hegarty B, Bossard P, Ferre P, Foufelle F. SREBP transcription factors: master regulators of lipid homeostasis. Biochimie. 2004;86(11):839–48. 10.1016/j.biochi.2004.09.018 . 15589694

[18] Hieb WF, Rothstein M. Sterol requirement for reproduction of a free-living nematode. Science. 1968;160(3829):778–80. 10.1126/science.160.3829.778 . 4869093

[19] McKay RM, McKay JP, Avery L, Graff JM. C elegans: a model for exploring the genetics of fat storage. Dev Cell. 2003;4(1):131–42. 10.1016/s1534-5807(02)00411-2 12530969 PMC4445237

[20] Osborne TF, Espenshade PJ. Evolutionary conservation and adaptation in the mechanism that regulates SREBP action: what a long, strange tRIP it's been. Genes Dev. 2009;23(22):2578–91. 10.1101/gad.1854309 19933148 PMC2779761

[21] Walker AK, Yang F, Jiang K, Ji JY, Watts JL, Purushotham A, et al Conserved role of SIRT1 orthologs in fasting-dependent inhibition of the lipid/cholesterol regulator SREBP. Genes Dev. 2010;24(13):1403–17. 10.1101/gad.1901210 20595232 PMC2895199

[22] Zhang L, Rajbhandari P, Priest C, Sandhu J, Wu X, Temel R, et al Inhibition of cholesterol biosynthesis through RNF145-dependent ubiquitination of SCAP. Elife. 2017;6 10.7554/eLife.28766 29068315 PMC5656429

[23] Cook EC, Nelson JK, Sorrentino V, Koenis D, Moeton M, Scheij S, et al Identification of the ER-resident E3 ubiquitin ligase RNF145 as a novel LXR-regulated gene. PLoS ONE. 2017;12(2):e0172721 10.1371/journal.pone.0172721 28231341 PMC5322959

[24] Jiang LY, Jiang W, Tian N, Xiong YN, Liu J, Wei J, et al Ring finger protein 145 (RNF145) is a ubiquitin ligase for sterol-induced degradation of HMG-CoA reductase. J Biol Chem. 2018;293(11):4047–55. 10.1074/jbc.RA117.001260 29374057 PMC5857978

[25] Bae YK, Barr MM. Sensory roles of neuronal cilia: cilia development, morphogenesis, and function in C. elegans. Front Biosci. 2008;13:5959–74. 10.2741/3129 18508635 PMC3124812

[26] Bargmann CI, Hartwieg E, Horvitz HR. Odorant-selective genes and neurons mediate olfaction in C. elegans. Cell. 1993;74(3):515–27. 10.1016/0092-8674(93)80053-h . 8348618

[27] Troemel ER, Kimmel BE, Bargmann CI. Reprogramming chemotaxis responses: sensory neurons define olfactory preferences in C. elegans. Cell. 1997;91(2):161–9. 10.1016/s0092-8674(00)80399-2 . 9346234

[28] Chow CY, Zhang Y, Dowling JJ, Jin N, Adamska M, Shiga K, et al Mutation of FIG 4 causes neurodegeneration in the pale tremor mouse and patients with CMT4J. Nature. 2007;448(7149):68–72. 10.1038/nature05876 17572665 PMC2271033

[29] Edenharter O, Schneuwly S, Navarro JA. Mitofusin-Dependent ER Stress Triggers Glial Dysfunction and Nervous System Degeneration in a Drosophila Model of Friedreich's Ataxia. Front Mol Neurosci. 2018;11:38 10.3389/fnmol.2018.00038 29563863 PMC5845754

[30] Ferguson CJ, Lenk GM, Meisler MH. Defective autophagy in neurons and astrocytes from mice deficient in PI(3,5)P2. Hum Mol Genet. 2009;18(24):4868–78. 10.1093/hmg/ddp460 19793721 PMC2778378

[31] Hoegg-Beiler MB, Sirisi S, Orozco IJ, Ferrer I, Hohensee S, Auberson M, et al Disrupting MLC1 and GlialCAM and ClC-2 interactions in leukodystrophy entails glial chloride channel dysfunction. Nat Commun. 2014;5:3475 10.1038/ncomms4475 . 24647135

[32] Lee YM, Sun YH. Maintenance of glia in the optic lamina is mediated by EGFR signaling by photoreceptors in adult Drosophila. PLoS Genet. 2015;11(4):e1005187 10.1371/journal.pgen.1005187 25909451 PMC4409299

[33] Muhlig-Versen M, da Cruz AB, Tschape JA, Moser M, Buttner R, Athenstaedt K, et al Loss of Swiss cheese/neuropathy target esterase activity causes disruption of phosphatidylcholine homeostasis and neuronal and glial death in adult Drosophila. J Neurosci. 2005;25(11):2865–73. 10.1523/JNEUROSCI.5097-04.2005 15772346 PMC1182176

[34] Delahaye JL, Foster OK, Vine A, Saxton DS, Curtin TP, Somhegyi H, et al Caenorhabditis elegans HOPS and CCZ-1 mediate trafficking to lysosome-related organelles independently of RAB-7 and SAND-1. Mol Biol Cell. 2014;25(7):1073–96. 10.1091/mbc.E13-09-0521 24501423 PMC3967972

[35] Demers-Lamarche J, Guillebaud G, Tlili M, Todkar K, Belanger N, Grondin M, et al Loss of Mitochondrial Function Impairs Lysosomes. J Biol Chem. 2016;291(19):10263–76. 10.1074/jbc.M115.695825 26987902 PMC4858975

[36] Morris C, Foster OK, Handa S, Peloza K, Voss L, Somhegyi H, et al Function and regulation of the Caenorhabditis elegans Rab32 family member GLO-1 in lysosome-related organelle biogenesis. PLoS Genet. 2018;14(11):e1007772 10.1371/journal.pgen.1007772 30419011 PMC6268011

[37] Ellis K, Bagwell J, Bagnat M. Notochord vacuoles are lysosome-related organelles that function in axis and spine morphogenesis. J Cell Biol. 2013;200(5):667–79. 10.1083/jcb.201212095 23460678 PMC3587825

[38] Takasuga S, Horie Y, Sasaki J, Sun-Wada GH, Kawamura N, Iizuka R, et al Critical roles of type III phosphatidylinositol phosphate kinase in murine embryonic visceral endoderm and adult intestine. Proc Natl Acad Sci U S A. 2013;110(5):1726–31. 10.1073/pnas.1213212110 23322734 PMC3562790

[39] Wada Y. Vacuoles in mammals: a subcellular structure indispensable for early embryogenesis. Bioarchitecture. 2013;3(1):13–9. 10.4161/bioa.24126 23572040 PMC3639239

[40] Ambrosio AL, Boyle JA, Di Pietro SM. Mechanism of platelet dense granule biogenesis: study of cargo transport and function of Rab32 and Rab38 in a model system. Blood. 2012;120(19):4072–81. 10.1182/blood-2012-04-420745 22927249 PMC3496959

[41] Bowman SL, Bi-Karchin J, Le L, Marks MS. The road to lysosome-related organelles: Insights from Hermansky-Pudlak syndrome and other rare diseases. Traffic. 2019;20(6):404–35. 10.1111/tra.12646 30945407 PMC6541516

[42] Cooper MS, Szeto DP, Sommers-Herivel G, Topczewski J, Solnica-Krezel L, Kang HC, et al Visualizing morphogenesis in transgenic zebrafish embryos using BODIPY TR methyl ester dye as a vital counterstain for GFP. Dev Dyn. 2005;232(2):359–68. 10.1002/dvdy.20252 . 15614774

[43] Elle IC, Olsen LC, Pultz D, Rodkaer SV, Faergeman NJ. Something worth dyeing for: molecular tools for the dissection of lipid metabolism in Caenorhabditis elegans. FEBS Lett. 2010;584(11):2183–93. 10.1016/j.febslet.2010.03.046 . 20371247

[44] O'Rourke EJ, Soukas AA, Carr CE, Ruvkun G. C. elegans major fats are stored in vesicles distinct from lysosome-related organelles. Cell Metab. 2009;10(5):430–5. 10.1016/j.cmet.2009.10.002 19883620 PMC2921818

[45] Yen K, Le TT, Bansal A, Narasimhan SD, Cheng JX, Tissenbaum HA. A comparative study of fat storage quantitation in nematode Caenorhabditis elegans using label and label-free methods. PLoS ONE. 2010;5(9). 10.1371/journal.pone.0012810 20862331 PMC2940797

[46] Sofroniew MV. Molecular dissection of reactive astrogliosis and glial scar formation. Trends Neurosci. 2009;32(12):638–47. 10.1016/j.tins.2009.08.002 19782411 PMC2787735

[47] Salama NR, Chuang JS, Schekman RW. Sec31 encodes an essential component of the COPII coat required for transport vesicle budding from the endoplasmic reticulum. Mol Biol Cell. 1997;8(2):205–17. 10.1091/mbc.8.2.205 9190202 PMC276074

[48] Skop AR, Liu H, Yates J 3rd, Meyer BJ, Heald R. Dissection of the mammalian midbody proteome reveals conserved cytokinesis mechanisms. Science. 2004;305(5680):61–6. 10.1126/science.1097931 15166316 PMC3679889

[49] Rolls MM, Hall DH, Victor M, Stelzer EH, Rapoport TA. Targeting of rough endoplasmic reticulum membrane proteins and ribosomes in invertebrate neurons. Mol Biol Cell. 2002;13(5):1778–91. 10.1091/mbc.01-10-0514 12006669 PMC111143

[50] Brockie PJ, Jensen M, Mellem JE, Jensen E, Yamasaki T, Wang R, et al Cornichons control ER export of AMPA receptors to regulate synaptic excitability. Neuron. 2013;80(1):129–42. 10.1016/j.neuron.2013.07.028 24094107 PMC3795439

[51] Liang B, Ferguson K, Kadyk L, Watts JL. The role of nuclear receptor NHR-64 in fat storage regulation in Caenorhabditis elegans. PLoS ONE. 2010;5(3):e9869 10.1371/journal.pone.0009869 20360843 PMC2845610

[52] Goldstein JL, DeBose-Boyd RA, Brown MS. Protein sensors for membrane sterols. Cell. 2006;124(1):35–46. 10.1016/j.cell.2005.12.022 . 16413480

[53] Brown MS, Goldstein JL. The SREBP pathway: regulation of cholesterol metabolism by proteolysis of a membrane-bound transcription factor. Cell. 1997;89(3):331–40. 10.1016/s0092-8674(00)80213-5 . 9150132

[54] Walker AK, Jacobs RL, Watts JL, Rottiers V, Jiang K, Finnegan DM, et al A conserved SREBP-1/phosphatidylcholine feedback circuit regulates lipogenesis in metazoans. Cell. 2011;147(4):840–52. 10.1016/j.cell.2011.09.045 22035958 PMC3384509

[55] Watts JL. Using Caenorhabditis elegans to Uncover Conserved Functions of Omega-3 and Omega-6 Fatty Acids. J Clin Med. 2016;5(2). 10.3390/jcm5020019 26848697 PMC4773775

[56] Watts JL, Browse J. A palmitoyl-CoA-specific delta9 fatty acid desaturase from Caenorhabditis elegans. Biochem Biophys Res Commun. 2000;272(1):263–9. 10.1006/bbrc.2000.2772 . 10872837

[57] Kahn-Kirby AH, Dantzker JL, Apicella AJ, Schafer WR, Browse J, Bargmann CI, et al Specific polyunsaturated fatty acids drive TRPV-dependent sensory signaling in vivo. Cell. 2004;119(6):889–900. 10.1016/j.cell.2004.11.005 . 15607983

[58] Bi X, Corpina RA, Goldberg J. Structure of the Sec23/24-Sar1 pre-budding complex of the COPII vesicle coat. Nature. 2002;419(6904):271–7. 10.1038/nature01040 . 12239560

[59] Bickford LC, Mossessova E, Goldberg J. A structural view of the COPII vesicle coat. Curr Opin Struct Biol. 2004;14(2):147–53. 10.1016/j.sbi.2004.02.002 . 15093828

[60] Witte K, Schuh AL, Hegermann J, Sarkeshik A, Mayers JR, Schwarze K, et al TFG-1 function in protein secretion and oncogenesis. Nat Cell Biol. 2011;13(5):550–8. 10.1038/ncb2225 21478858 PMC3311221

[61] Matsuda M, Korn BS, Hammer RE, Moon YA, Komuro R, Horton JD, et al SREBP cleavage-activating protein (SCAP) is required for increased lipid synthesis in liver induced by cholesterol deprivation and insulin elevation. Genes Dev. 2001;15(10):1206–16. 10.1101/gad.891301 11358865 PMC313801

[62] Kim DH, Sarbassov DD, Ali SM, King JE, Latek RR, Erdjument-Bromage H, et al mTOR interacts with raptor to form a nutrient-sensitive complex that signals to the cell growth machinery. Cell. 2002;110(2):163–75. 10.1016/s0092-8674(02)00808-5 . 12150925

[63] Laplante M, Sabatini DM. mTOR signaling in growth control and disease. Cell. 2012;149(2):274–93. 10.1016/j.cell.2012.03.017 22500797 PMC3331679

[64] Wullschleger S, Loewith R, Hall MN. TOR signaling in growth and metabolism. Cell. 2006;124(3):471–84. 10.1016/j.cell.2006.01.016 . 16469695

[65] Shimano H, Horton JD, Hammer RE, Shimomura I, Brown MS, Goldstein JL. Overproduction of cholesterol and fatty acids causes massive liver enlargement in transgenic mice expressing truncated SREBP-1a. J Clin Invest. 1996;98(7):1575–84. 10.1172/JCI118951 8833906 PMC507590

[66] Porstmann T, Santos CR, Griffiths B, Cully M, Wu M, Leevers S, et al SREBP activity is regulated by mTORC1 and contributes to Akt-dependent cell growth. Cell Metab. 2008;8(3):224–36. 10.1016/j.cmet.2008.07.007 18762023 PMC2593919

[67] Narumiya S, Ohno K, Fujiwara M, Fukushima M. Site and mechanism of growth inhibition by prostaglandins. II. Temperature-dependent transfer of a cyclopentenone prostaglandin to nuclei. J Pharmacol Exp Ther. 1986;239(2):506–11. . 3772805

[68] Straus DS, Pascual G, Li M, Welch JS, Ricote M, Hsiang CH, et al 15-deoxy-delta 12,14-prostaglandin J2 inhibits multiple steps in the NF-kappa B signaling pathway. Proc Natl Acad Sci U S A. 2000;97(9):4844–9. 10.1073/pnas.97.9.4844 10781090 PMC18320

[69] Liu JJ, Green P, John Mann J, Rapoport SI, Sublette ME. Pathways of polyunsaturated fatty acid utilization: implications for brain function in neuropsychiatric health and disease. Brain Res. 2015;1597:220–46. 10.1016/j.brainres.2014.11.059 25498862 PMC4339314

[70] Luchtman DW, Song C. Cognitive enhancement by omega-3 fatty acids from child-hood to old age: findings from animal and clinical studies. Neuropharmacology. 2013;64:550–65. 10.1016/j.neuropharm.2012.07.019 . 22841917

[71] Yehuda S. Omega-6/omega-3 ratio and brain-related functions. World Rev Nutr Diet. 2003;92:37–56. 10.1159/000073791 . 14579682

[72] Zhang W, Li P, Hu X, Zhang F, Chen J, Gao Y. Omega-3 polyunsaturated fatty acids in the brain: metabolism and neuroprotection. Front Biosci (Landmark Ed). 2011;16:2653–70. 10.2741/3878 . 21622201

[73] Krycer JR, Sharpe LJ, Luu W, Brown AJ. The Akt-SREBP nexus: cell signaling meets lipid metabolism. Trends Endocrinol Metab. 2010;21(5):268–76. 10.1016/j.tem.2010.01.001 . 20117946

[74] Brenner S. The genetics of Caenorhabditis elegans. Genetics. 1974;77(1):71–94. 4366476 10.1093/genetics/77.1.71 PMC1213120

[75] Schindelin J, Arganda-Carreras I, Frise E, Kaynig V, Longair M, Pietzsch T, et al Fiji: an open-source platform for biological-image analysis. Nat Methods. 2012;9(7):676–82. 10.1038/nmeth.2019 22743772 PMC3855844

[76] Altun ZF, Hall DH. Handbook of C. elegans Anatomy. 2020 In: WormAtlas [Internet]. Available from: http://www.wormatlas.org/hermaphrodite/hermaphroditehomepage.htm.

[77] Ward S. Chemotaxis by the nematode Caenorhabditis elegans: identification of attractants and analysis of the response by use of mutants. Proc Natl Acad Sci U S A. 1973;70(3):817–21. 10.1073/pnas.70.3.817 4351805 PMC433366

[78] Deline ML, Vrablik TL, Watts JL. Dietary supplementation of polyunsaturated fatty acids in Caenorhabditis elegans. J Vis Exp. 2013;(81). 10.3791/50879 24326396 PMC3992124

[79] Guan Z, Li S, Smith DC, Shaw WA, Raetz CR. Identification of N-acylphosphatidylserine molecules in eukaryotic cells. Biochemistry. 2007;46(50):14500–13. 10.1021/bi701907g 18031065 PMC2535763

[80] Heppert JK, Dickinson DJ, Pani AM, Higgins CD, Steward A, Ahringer J, et al Comparative assessment of fluorescent proteins for in vivo imaging in an animal model system. Mol Biol Cell. 2016;27(22):3385–94. 10.1091/mbc.E16-01-0063 27385332 PMC5221575

