# Supplementary figures and images for: Regulation of glial size by eicosapentaenoic acid through a novel Golgi apparatus mechanism
Source: PLoS Biol. 2020 Dec 28;18(12):e3001051. doi: 10.1371/journal.pbio.3001051 (PMC7793280; doi:10.1371/journal.pbio.3001051)

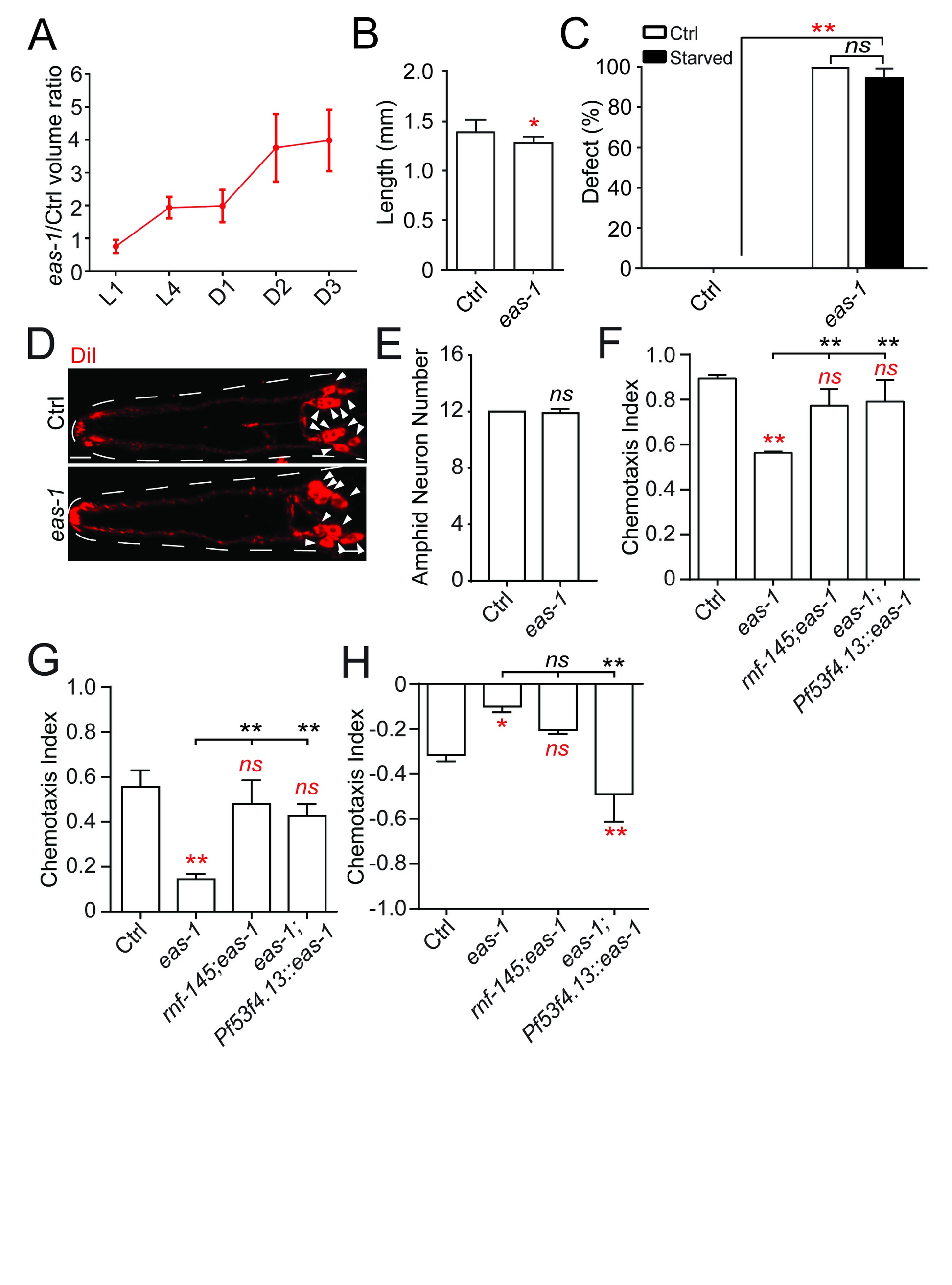

Supplement: S1 Fig — (A) The ratio of eas-1(yad70) to WT AMsh cell body volume at each respective stage measured. (B) The total length of D2 WT and eas-1(yad70) animals. Each bar represents at least 10 worms. (C) The percentage of D2 WT or eas-1(yad70) animals with enlarged AMsh cells after food deprivation since L4. Data sets comprised of control unstarved worms are filled white, while those comprised of starved filled are colored black. Each bar represents 3 experiments of 50 worms each. Two-way ANOVA, followed by Tukey HSD test, *p < 0.05, **p < 0.01. (D) DiI staining of amphid neurons in WT or eas-1(yad70) D1 adult animals. Arrowheads point to each visible amphid neuron cell body. As these 12 neurons are close to each other, in the projection images, 2 neurons are difficult to present, but we clearly observed 12 neurons under the microscope. Scale bar, 10 μm. White dotted lines outline the worm. (E) Number of amphid neurons stained by DiI in WT or eas-1(yad70) D1 adult animals. One-sample t test, *p < 0.05, **p < 0.01. (F–H) Chemotaxis indexes of WT, eas-1(yad70), rnf-145(yad110);eas-1(yad70), and eas-1(yad70);Pf53f4.13::eas-1 animals in response to 0.5% benzaldehyde (F), 1% pyrazine (G), or 100% 1-octanol (H). Each bar represents 3 experiments of at least 100 animals. One-way ANOVA, followed by Tukey HSD test, *p < 0.05, **p < 0.01. Underlying data for graphs can be found in S1 Data. AMsh, amphid sheath; ANOVA, analysis of variance; D2, day 2; HSD, honestly significant difference; WT, wild-type. (TIF) [file pbio.3001051.s001.tif]

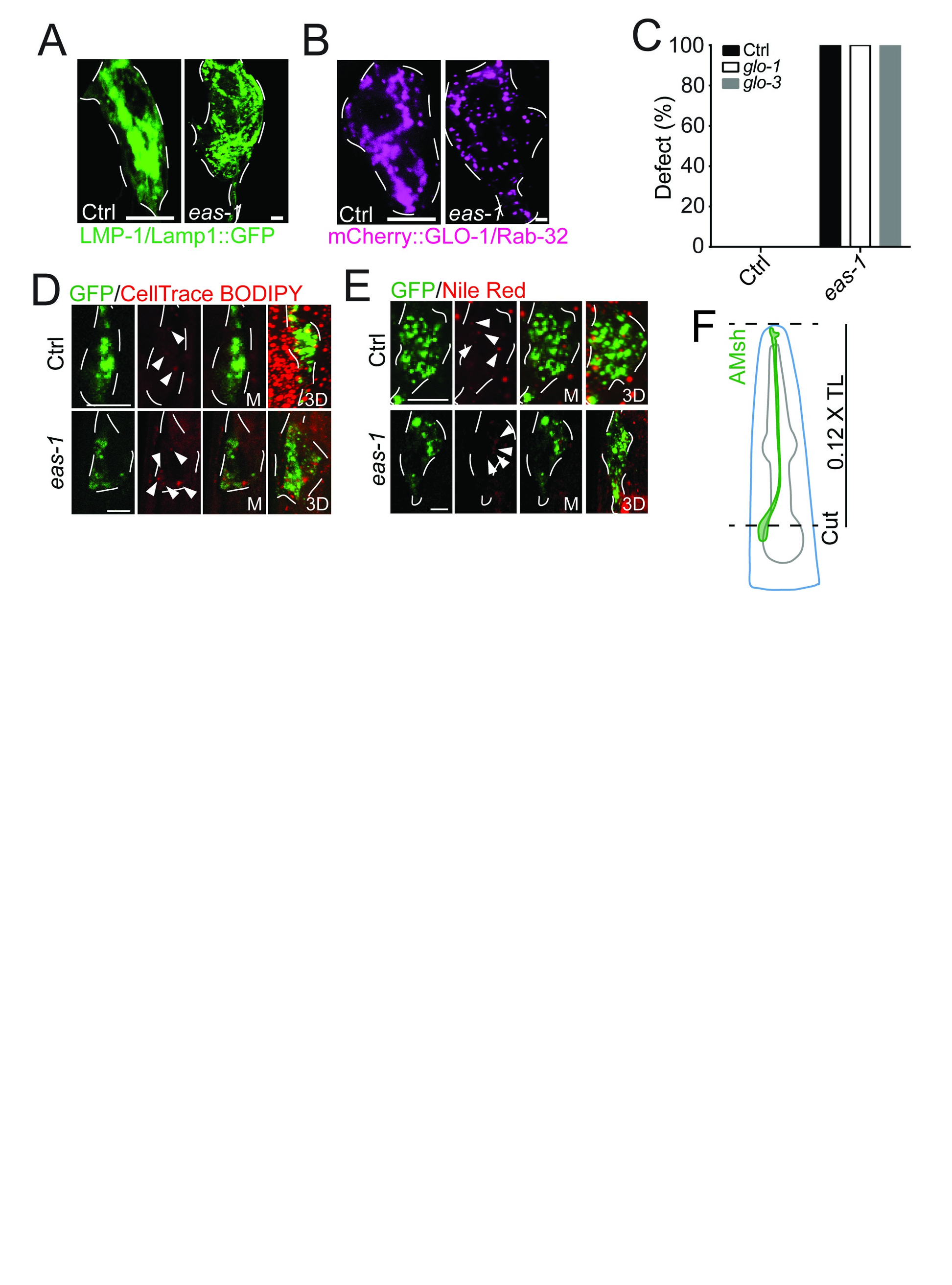

Supplement: S2 Fig — (A) Expression of the lysosomal membrane fusion protein LMP-1::GFP in the AMsh of D1 adult control and eas-1(yad83) animals. Scale bar, 10 μm. (B) Expression of the fusion reporter mCherry::GLO-1 in the AMsh of D1 adult control and eas-1(yad83) animals. (C) Percentage of D2 animals with enlarged AMsh cells after RNAi knockdown of control, glo-1 or glo-3 in either WT or eas-1(yad70) backgrounds. Each point represents 3 experiments of at least 50 animals. (D, E) Staining of PFA-fixed D2 adult animals with (D) CellTrace BODIPY TR or (E) Nile Red in control and eas-1(yad70) backgrounds. White arrowheads point to dye puncta within the AMsh cell. Left 3 panels are from a single focus plane: M, merged; 3D, 3D reconstruction. Scale bar, 10 μm. (F) Schematic of where TEM cross-sections were cut to show the AMsh cell bodies. TL, the measured total length from the nose to tail. Sections were cut in the region around 0.12 total lengths from the nose tip and imaged by TEM. Scale bar, 10 μm. White dotted lines outline the AMsh cell body. Data are represented as mean ± SD. Underlying data for graphs can be found in S1 Data. AMsh, amphid sheath; D1, day 1; D2, day 2; LRO, lysosome-related organelle; RNAi, RNA interference; SD, standard deviation; TEM, transmission electron microscopy; WT, wild-type. (TIF) [file pbio.3001051.s002.tif]

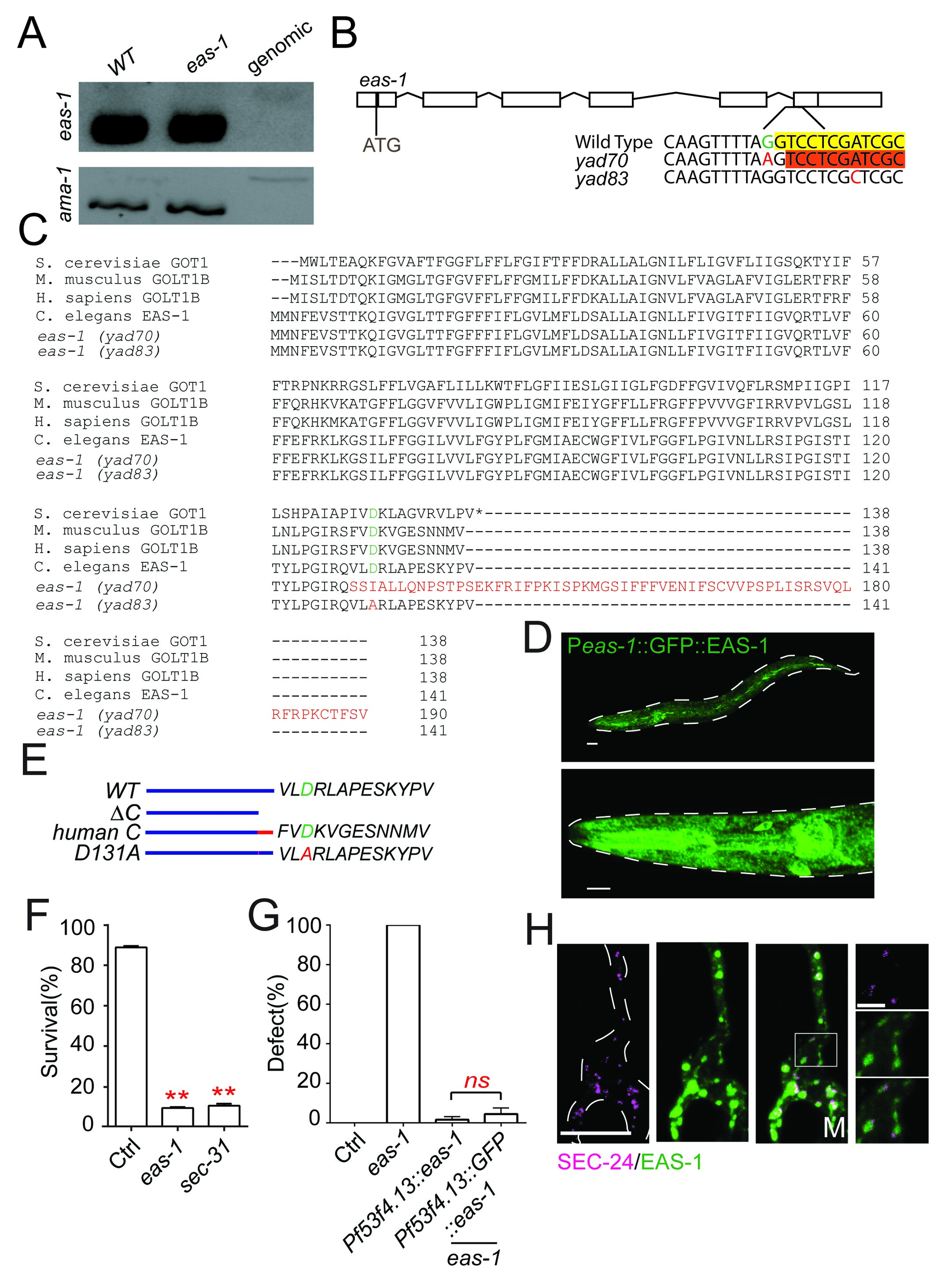

Supplement: S3 Fig — (A) RT-PCR products of eas-1 in WT and eas-1(yad70) mutants. ama-1 was used as a housekeeping gene, and the right column contains PCR fragments amplified from WT genomic DNA. (B) Genomic organization of eas-1. The point mutations in the yad70 and yad83 alleles are colored red. Part of the WT and yad70 exons after the point mutation site are highlighted. (C) The amino acid sequences of eas-1 and its homologs in Saccharomyces cerevisiae, Mus musculus, and Homo sapiens. The yad70 and yad83 alleles are also included. The conserved D131 residue is colored green, while differing residues in the alleles are colored red. (D) Expression of the Peas-1::GFP::eas-1 translational reporter in L1 (top panel) and D1 animals (bottom panel). Dashed lines outline the shape of the worm. Scale bar, 10 μm. (E) Schematic of different eas-1 constructs from Fig 2B—WT eas-1, eas-1 lacking the carboxyl terminus (ΔC), eas-1 with the D131A substitution (D131A), and eas-1 with a human carboxyl terminus (human C). (F) Percentage survival of WT animals after RNAi knockdown of control, eas-1, and sec-31. Embryos were plated and the percentage of worms that successfully hatched were quantified. (G) Rescue experiments using either Pf53f4.13::eas-1 or Pf53f4.13::GFP::eas-1 in D2. Genetic background is WT unless otherwise indicated. (H) Confocal images of the fusion reporter GFP::EAS-1 and the COPII vesicle marker SEC-24::mCherry in the AMsh of D1 adults. Scale bar, 10 μm for top figures, 2 μm for zoomed in figures below. White dotted lines outline the AMsh cell body. Data are represented as mean ± SD. One-way ANOVA, followed by Tukey HSD test, *p < 0.05, **p < 0.01. Each point represents 3 experiments of at least 50 animals. Underlying data for graphs can be found in S1 Data. AMsh, amphid sheath; ANOVA, analysis of variance; D1, day 1; D2, day 2; HSD, honestly significant difference; RNAi, RNA interference; RT-PCR, reverse transcription PCR; SD, standard deviation; WT, wild-type. (TIF) [file pbio.3001051.s003.tif]

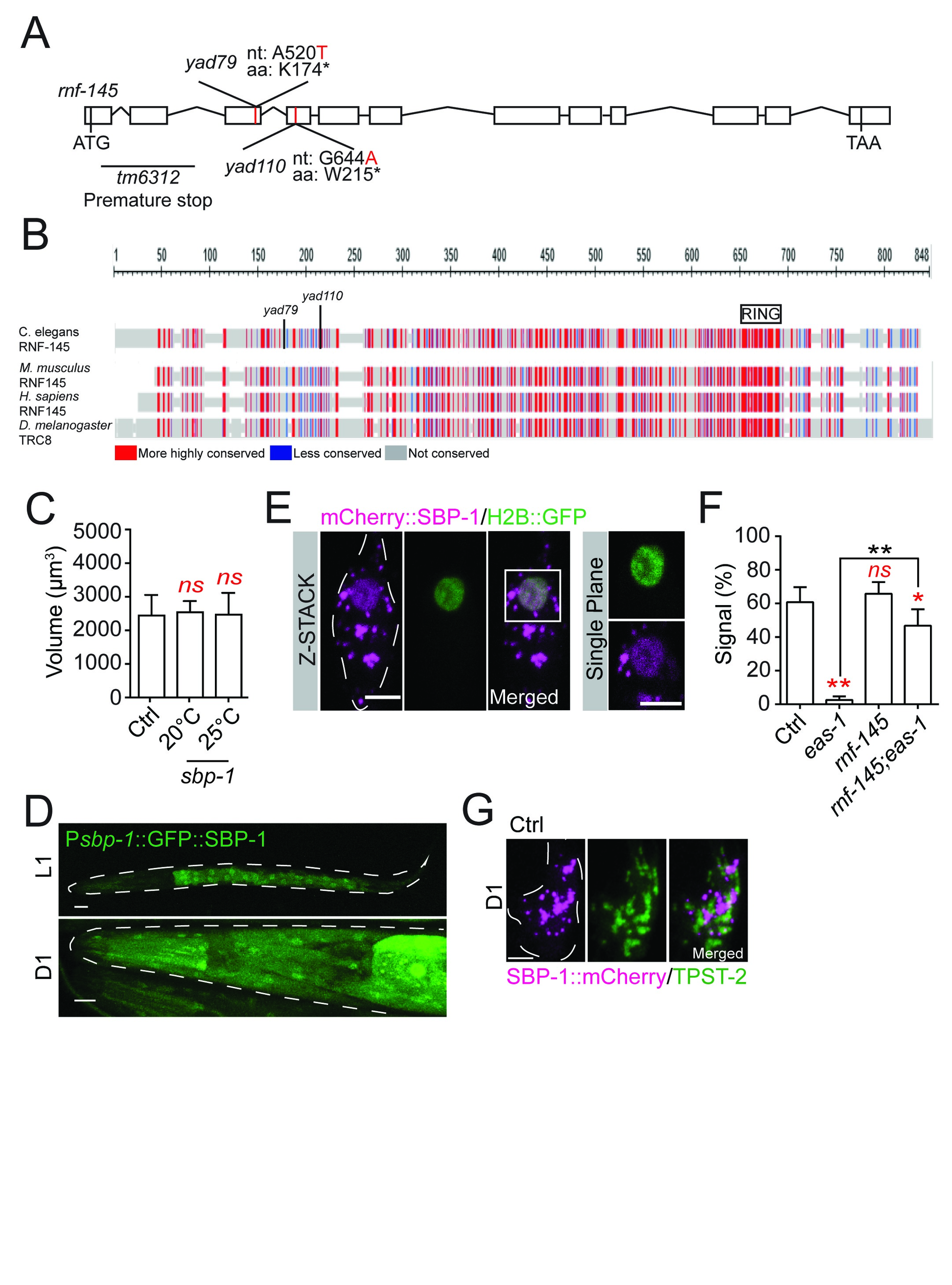

Supplement: S4 Fig — (A) Genomic organization of rnf-145 on top, with the point mutations of the yad79 and yad110 alleles labeled in red and the span of deletion of the tm6312 allele denoted with a black line. (B) The predicted amino acid structure of rnf-145 and its homologs in Mus musculus, Homo sapiens, and Drosophila melanogaster. More highly conserved regions are labeled in red. (C) The volumes of AMsh cell bodies in WT and sbp-1(ep79) animals in μm3. sbp-1(ep79) mutants are temperature sensitive, and after hatching were cultured at the permissive 20°C and the nonpermissive 25°C, respectively, before imaging. Each point represents at least 10 animals. (D) Expression of the Psbp-1::GFP::sbp-1 translational reporter in L1 (top panel) and D1 animals (bottom panel). Dashed lines outline the shape of the worm. (E) Coexpression of mCherry::SBP-1 and the nuclear marker H2B::GFP in the AMsh cells of WT D1 adults. White dotted lines outline the AMsh cell body. (F) Quantification of the percentage total signal of mCherry::SBP-1 signal that colocalizes with H2B::GFP. Each bar represents quantification of 6 worms. (G) Coexpression of SBP-1::mCherry and the trans-Golgi marker GFP::TSPT-2 in the AMsh cells of WT D1 adults. White dotted lines outline the AMsh cell body. Scale bar, 10 μm. Data are represented as mean ± SD. Student t test, *p < 0.05, **p < 0.01. Underlying data for graphs can be found in S1 Data. AMsh, amphid sheath; D1, day 1; SD, standard deviation; WT, wild-type. (TIF) [file pbio.3001051.s004.tif]

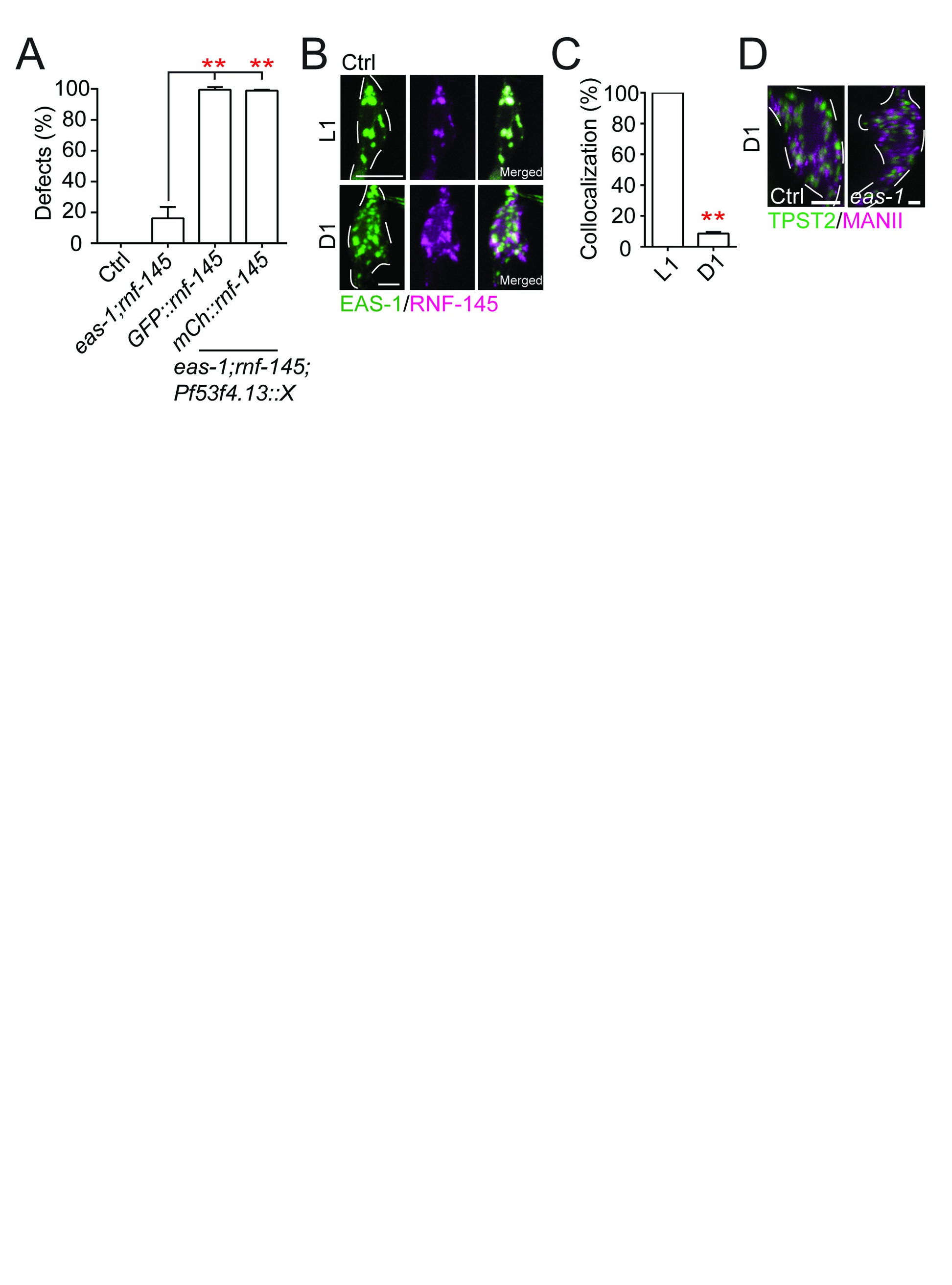

Supplement: S5 Fig — (A) Rescue of rnf-145(yad110);eas-1(yad70) using the fusion reporters Pf53f4.13::GFP::rnf-145 and Pf53f4.13::mCherry::rnf-145. Data are represented as mean ± SD. One-way ANOVA, followed by Tukey HSD test, *p < 0.05, **p < 0.01. Each point represents 3 experiments of at least 50 animals. (B) Coexpression of GFP::EAS-1 with mCherry::RNF-145 in the AMsh cells of WT animals. Animals were imaged at L1 and D1 adult stages. Scale bar, 10 μm. (C) Proportion of mCherry::RNF-145 puncta that colocalize with GFP::EAS-1 puncta in during the L1 and D1 stages. Data are represented as mean ± SD. Student t test, *p < 0.05, **p < 0.01. At least 10 animals were quantified for each condition. (D) Coexpression of TPST-2::GFP and mRuby::MannII in AMsh cells of control and eas-1(yad70) D1 adult animals. Scale bar, 10 μm. White dotted lines outline the AMsh cell body. Underlying data for graphs can be found in S1 Data. AMsh, amphid sheath; ANOVA, analysis of variance; D1, day 1; HSD, honestly significant difference; SD, standard deviation; WT, wild-type. (TIF) [file pbio.3001051.s005.tif]

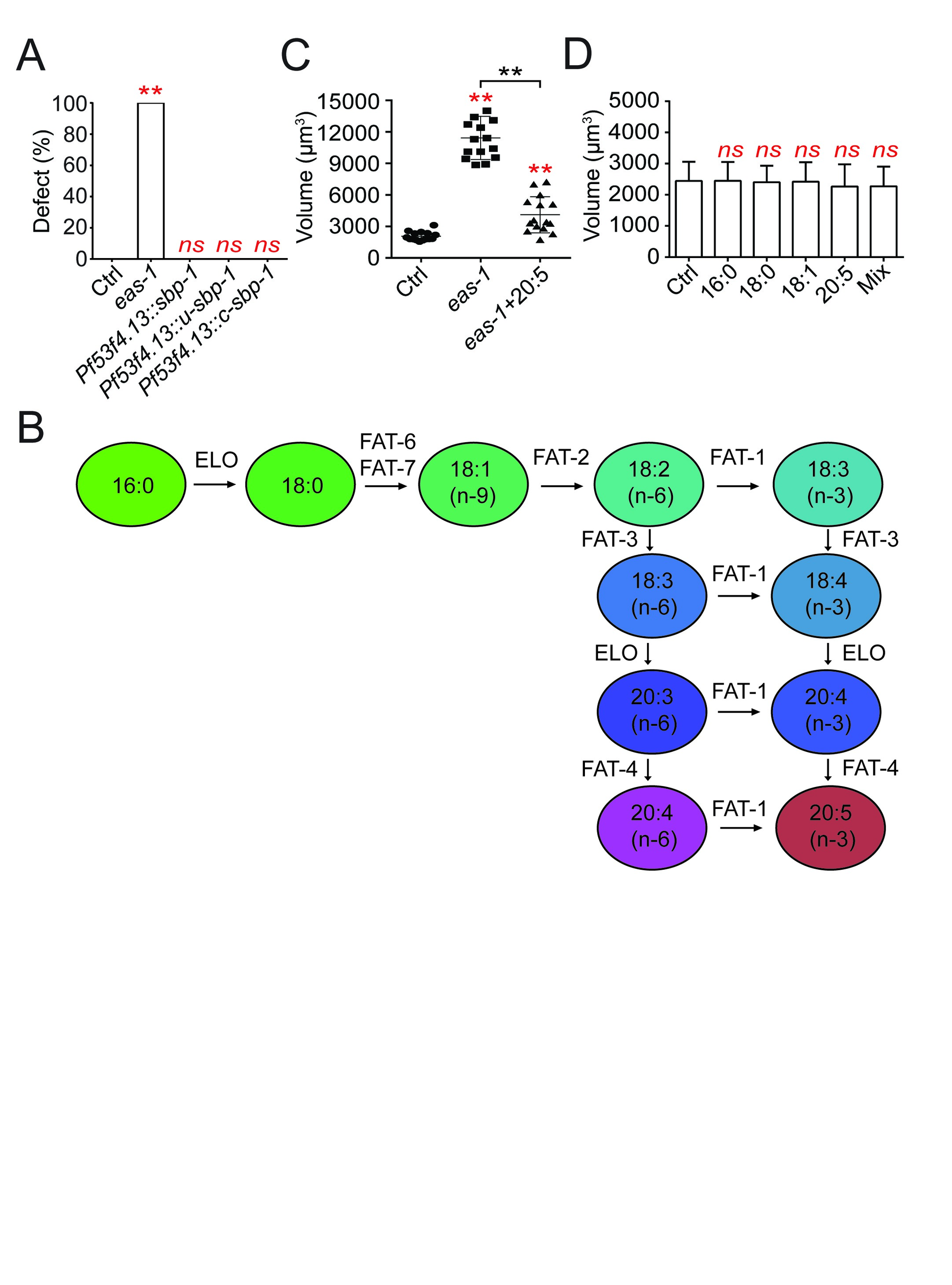

Supplement: S6 Fig — (A) Percentage of animals with enlarged AMsh cells after ectopic expression of the different sbp-1 constructs in the AMsh cells of D2 WT animals. Each point represents 3 experiments of at least 50 animals. (B) Schematic of the LC-PUFA synthesis pathway in C. elegans. (C) The AMsh cell body volumes of D2 control, eas-1(yad70) and eas-1(yad70) supplemented with 200 μM EPA. Fifteen D2 animals were randomly picked quantified for each condition. (D) The AMsh cell body volumes of D2 WT animals supplemented with dietary polyunsaturated fatty acids at a concentration of 200 μm each. Mix represents a mixture of all the above fatty acids at a concentration of 200 μm each. Each bar represents at least 10 animals. Data are represented as mean ± SD. One-way ANOVA, followed by Tukey HSD test, *p < 0.05, **p < 0.01. Underlying data for graphs can be found in S1 Data. AMsh, amphid sheath; ANOVA, analysis of variance; D2, day 2; EPA, eicosapentaenoic acid; HSD, honestly significant difference; LC-PUFA, long-chain polyunsaturated fatty acid; SD, standard deviation; WT, wild-type. (TIF) [file pbio.3001051.s006.tif]

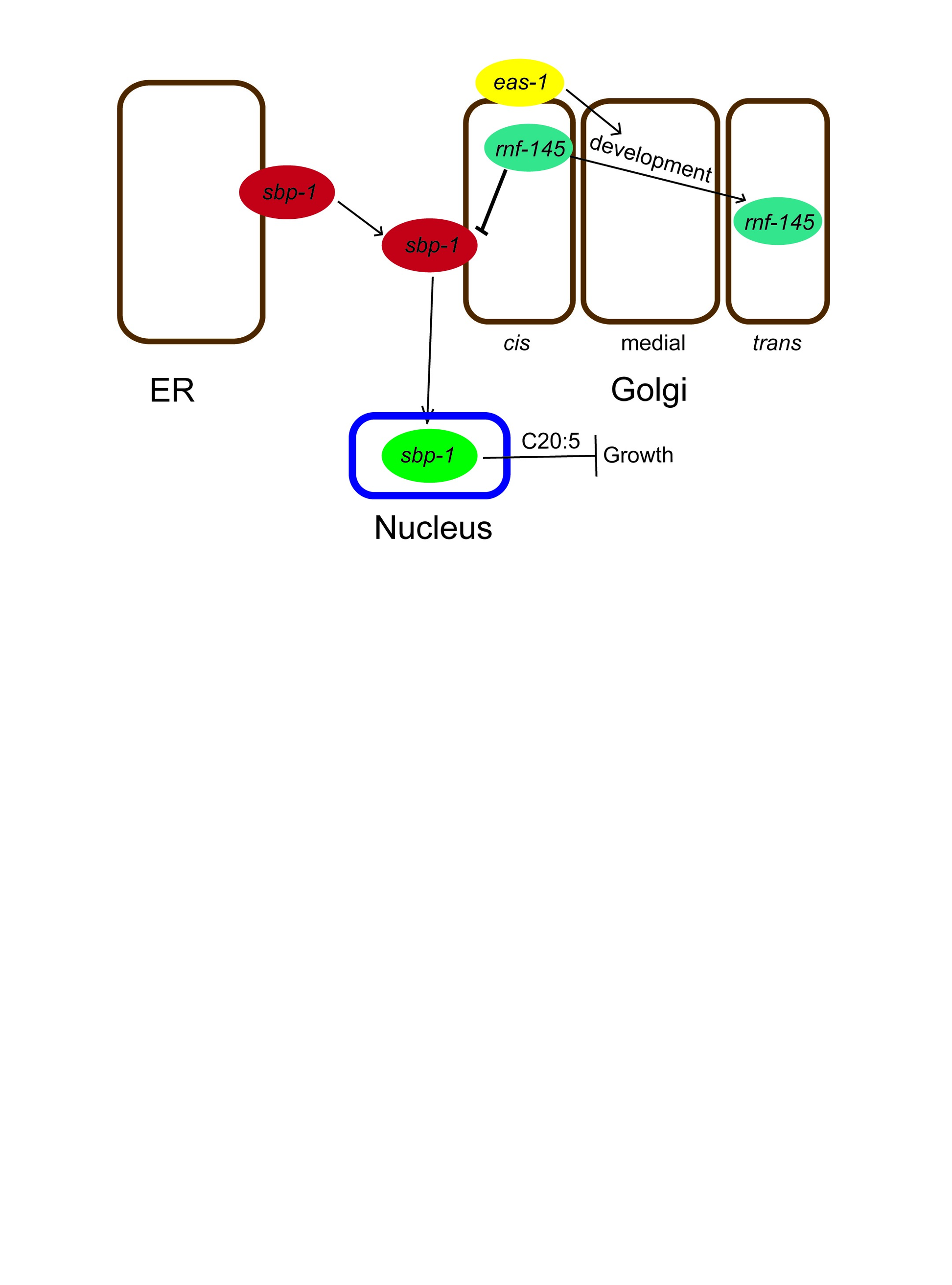

Supplement: S7 Fig — Model of how eas-1/GOLT1B may regulate growth through release of inhibition on sbp-1/SREBP. (TIF) [file pbio.3001051.s007.tif]

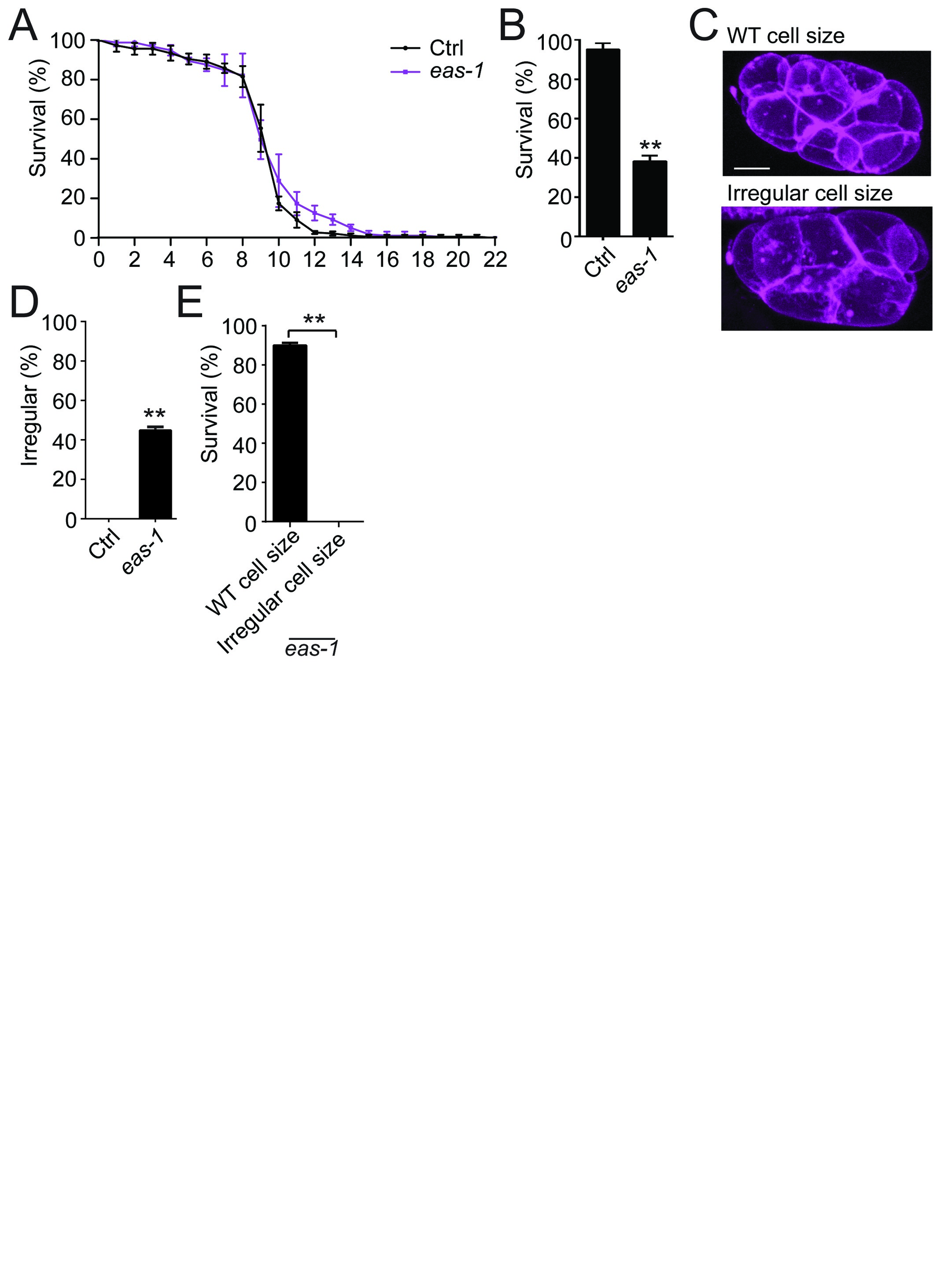

Supplement: S8 Fig — (A) Percentage of control (black) or eas-1(yad70) (magenta) animals surviving at each time point. Three replicates of at least 50 worms were quantified for each time point. (B) Percentage of control and eas-1(yad70) animals that survive past the embryonic stage. Each bar represents 3 experiments of at least 60 embryos. (C) eas-1(yad70) embryos that exhibit WT-like (shown top) and irregular (bottom) cell sizes at 20°C. Labeled by the Pmex-5::mCherry-C1::PLC(delta)-PH::tbb-2 3'UTR reporter that labels all cell membranes in the embryo. Scale bar, 10 μm. (D) Percentage of eas-1(yad70) embryos with irregular cell sizes. (E) Percentage survival of eas-1(yad70) animals past embryogenesis based on whether they start with irregular cell sizes. Each bar represents 3 experiments of at least 50 embryos. Data are represented as mean ± SD. Student t test, *p < 0.05, **p < 0.01. Underlying data for graphs can be found in S1 Data. SD, standard deviation; WT, wild-type. (TIF) [file pbio.3001051.s008.tif]
