# Supplementary material for: Regulation of glial size by eicosapentaenoic acid through a novel Golgi apparatus mechanism
Source: PLoS Biol. 2020 Dec 28;18(12):e3001051. doi: 10.1371/journal.pbio.3001051 (PMC7793280; doi:10.1371/journal.pbio.3001051)
Supplement: S1 Table — Table of strains and plasmids used in this study. (DOCX) [file pbio.3001051.s009.docx]

Supplemental Table 1, Strain list

| Strain | Genotype | Plasmid | Related Figures |
| --- | --- | --- | --- |
| NYL1269 | *Pf53f4.13::GFP Posm-10::mCherry, Pttx-3::RFP*  *(yadIs48)* | PNYL1124 | Fig 1 and  WT control for other figures |
| NYL1326 | *Pf53f4.13::GFP Posm-10::mCherry, Pttx-3::RFP*  *(yadIs48) ; eas-1(yad70)* |  | Fig 1 and *eas-1* control for other figures |
| NYL1444 | *eas-1(yad70) II ; Pf53f4.13::GFP Posm-*  *10::mCherry, Pttx-3::RFP (yadIs48) ; Peas-1::eas1, Pttx-3::RFP(yadEx707)* | PCR  fragment | Fig 2A |
| NYL1445 | *eas-1(yad70) II ; Pf53f4.13::GFP Posm-*  *10::mCherry, Pttx-3::RFP (yadIs48) ; Peas-1::eas1, Pttx-3::RFP (yadEx708)* | PCR  fragment | Fig 2A |
| NYL1446 | *eas-1(yad70) II ; Pf53f4.13::GFP Posm-*  *10::mCherry, Pttx-3::RFP (yadIs48) ; Pdpy-*  *30::eas-1, Pttx-3::RFP(yadEx709)* | PNYL1125 | Fig 2A |
| NYL1447 | *eas-1(yad70) II ; Pf53f4.13::GFP Posm-*  *10::mCherry, Pttx-3::RFP (yadIs48) ; Pdpy-*  *30::eas-1, Pttx-3::RFP(yadEx710)* | PNYL1125 | Fig 2A |
| NYL1448 | *eas-1(yad70) II ; Pf53f4.13::GFP Posm-*  *10::mCherry, Pttx-3::RFP (yadIs48) ;*  *Pf53f4.13::eas-1, Pttx-3::RFP(yadEx711)* | PNYL1126 | Fig 2A, 2D; S1F, S1G, S1H and S3G Fig |
| NYL1449 | *eas-1(yad70) II ; Pf53f4.13::GFP Posm-*  *10::mCherry, Pttx-3::RFP (yadIs48) ;*  *Pf53f4.13::eas-1, Pttx-3::RFP(yadEx712)* | PNYL1126 | Fig 2A, 2D; 1f, S1G, S1H and S3G Fig |
| NYL1536 | *eas-1(yad70) II Pf53f4.13::GFP Posm-10::mCherry,*  *Pttx-3::RFP (yadIs48) ; Pf53f4.13::GOT1B(human cDNA), Pttx-3::RFP(yadEx779)* | PNYL1127 | Fig 2A |
| NYL1537 | *eas-1(yad70) II Pf53f4.13::GFP Posm-10::mCherry,*  *Pttx-3::RFP (yadIs48) ; Pf53f4.13::GOT1B(human cDNA), Pttx-3::RFP(yadEx780)* | PNYL1127 | Fig 2A |
| NYL1686 | *eas-1(yad83) II; Pf53f4.13::GFP, Posm-10::mCherry (yadIs48)* |  | Fig 2A |
| NYL1540 | *eas-1(yad70) II Pf53f4.13::GFP Posm-10::mCherry,*  *Pttx-3::RFP (yadIs48) ; Pf53f4.13::eas-1 (Cterminal deletion), Pttx-3::RFP(yadEx783)* | PNYL1128 | Fig 2B |
| NYL1541 | *eas-1(yad70) II Pf53f4.13::GFP Posm-10::mCherry,*  *Pttx-3::RFP (yadIs48) ; Pf53f4.13::eas-1 (Cterminal deletion), Pttx-3::RFP(yadEx784)* | PNYL1128 | Fig 2B |
| NYL1538 | *eas-1(yad70) II Pf53f4.13::GFP Posm-10::mCherry,*  *Pttx-3::RFP (yadIs48) ; Pf53f4.13::eas-1 (human C), Pttx3::RFP(yadEx781)* | PNYL1129 | Fig 2B |
| NYL1539 | *eas-1(yad70) II Pf53f4.13::GFP Posm-10::mCherry,*  *Pttx-3::RFP (yadIs48) ; Pf53f4.13::eas-1 (human C), Pttx3::RFP(yadEx782)* | PNYL1129 | Fig 2B |
| NYL1482 | *Pf53f4.13::GFP::eas-1 , Pf53f4.13::mRuby::ManII,*  *Pttx-3::RFP(yadEx742)* | PNYL1131, PNYL1132 | Fig 2E |
| NYL1483 | *Pf53f4.13::GFP::eas-1 , Pf53f4.13::mRuby::ManII,*  *Pttx-3::RFP(yadEx743)* | PNYL1131, PNYL1132 | Fig 2E |
| NYL1678 | *Pf53f4.13::GFP::eas-1; Pf53f4.13::mCherry:eas-1(D131A)(yadEx861)* | PNYL1311,  PNYL1132 | Fig 2F |
| NYL1909 | *eas-1(yad70) II Pf53f4.13::GFP Posm-10::mCherry,*  *Pttx-3::RFP (yadIs48) ; Phsp::eas-1, Pttx3::GFP(yadEx963)* | PNYL1133 | Fig 2G |
| NYL1910 | *eas-1(yad70) II Pf53f4.13::GFP Posm-10::mCherry,*  *Pttx-3::RFP (yadIs48) ; Phsp::eas-1, Pttx3::GFP(yadEx964)* | PNYL1133 | Fig 2G |
| NYL1612 | *eas-1(yad70) II Pf53f4.13::GFP Posm-10::mCherry, Pttx-3::RFP (yadIs48) ; rnf-145 (yad79)* |  | Fig 3A |
| NYL2007 | *rnf-145 (yad110) ; eas-1(yad70) II Pf53f4.13::GFP Posm-10::mCherry, Pttx-3::RFP (yadIs48) II* |  | Fig 3A – 3D, 5D, 6E; S1F, S1G, S1H and S5A Fig |
| NYL1970 | *rnf-145 (tm6312) I ; eas-1(yad70) II Pf53f4.13::GFP Posm-10::mCherry, Pttx-3::RFP (yadIs48)* |  | Fig 3A |
| NYL2273 | *rnf-145 (yad110) ; Pf53f4.13::GFP Posm10::mCherry, Pttx-3::RFP (yadIs48) II* |  | Fig 3B, 5C and 6E |
| NYL2129 | *Prnf-145::rnf-145, Pttx-3::GFP(yadEx1087) ; rnf145 (yad110); eas-1(yad70) II Pf53f4.13::GFP Posm-10::mCherry, Pttx-3::RFP (yadIs48) II* | PCR  fragment | Fig 3C |
| NYL2130 | *Prnf-145::rnf-145, Pttx-3::GFP(yadEx1088) ; rnf145 (yad110); eas-1(yad70) II Pf53f4.13::GFP Posm-10::mCherry, Pttx-3::RFP (yadIs48) II* | PCR  fragment | Fig 3C |
| NYL2131 | *Pf53f4.13::rnf-145, Pttx-3::GFP(yadEx1089) ; rnf145 (yad110); eas-1(yad70) II Pf53f4.13::GFP Posm-10::mCherry, Pttx-3::RFP (yadIs48) II* | PNYL1134 | Fig 3C |
| NYL2132 | *Pf53f4.13::rnf-145, Pttx-3::GFP(yadEx1090) ; rnf145 (yad110); eas-1(yad70) II Pf53f4.13::GFP Posm-10::mCherry, Pttx-3::RFP (yadIs48) II* | PNYL1134 | Fig 3C |
| NYL2207 | *sbp-1(ep79) III ; Pf53f4.13::GFP Posm10::mCherry, Pttx-3::RFP (yadIs48) II* |  | Fig 3D; S4C Fig |
| NYL1844 | *Pf53f4.13::GFP Posm-10::mCherry, Pttx-3::RFP (yadIs48) ; eas-1(yad70) II ; sbp-1(ep79) III* |  | Fig 3D |
| NYL2269 | *sbp-1(ep79) III ; eas-1(yad70) II ; rnf-145(yad110)*  *V ; Pf53f4.13::GFP Posm-10::mCherry (yadIs48) II* |  | Fig 3D |
| NYL2180 | *Pf53f4.13::GFP Posm-10::mCherry, Pttx-3::RFP*  *(yadIs48) II ;Pf53f4.13::mCherry::sbp-1, Pttx3::GFP(yadEx1136)* | PNYL1135 | Fig 3E |
| NYL2183 | *eas-1(yad70) II Pf53f4.13::GFP Posm-10::mCherry,*  *Pttx-3::RFP (yadIs48) II ; Pf53f4.13::mCherry::sbp-1, Pttx-3::GFP(yadEx1136)* | PNYL1135 | Fig 3E |
| NYL2256 | *rnf-145(yad110) V ; Pf53f4.13::mCherry::sbp-1, Pttx3::GFP(yadEx1136)* | PNYL1135 | Fig 3E |
| NYL2184 | *eas-1(yad70) II Pf53f4.13::GFP Posm-10::mCherry, Pttx-3::RFP (yadIs48) II ; rnf-145 (yad110);*  *Pf53f4.13::mCherry::sbp-1, Pttx-3::GFP(yadEx1136)* | PNYL1135 | Fig 3E |
| NYL2405 | *Pf53f4.13::mCherry::sbp-1; Pf53f4.13::GFP::eas-1(yadEx1239)* | PNYL1135,  PNYL1132 | Fig 3F and 3H |
| NYL2429 | *Pf53f4.13::sbp-1::mCherry; Pf53f4.13::GFP::eas-1(yadEx1252)* | PNYL1265,  PNYL1132 | Fig 3G and 3H |
| NYL2518 | *Pf53f4.13::GFP::rnf-145; Pf53f4.13::mRuby::MANII(yadEx1298)* | PNYL1266,  PNYL1131 | Fig 4A and 4E |
| NYL2416 | *Pf53f4.13::TPST-2::EGFP; Pf53f4.13::mCherry::rnf-145(yadEx1244)* | PNYL1267,  PNYL1268 | Fig 4B and 4F |
| NYL2522 | *eas-1(yad83) II ; Pf53f4.13::GFP::rnf-145; Pf53f4.13::mRuby::MANII(yadEx1298)* | PNYL1266,  PNYL1131 | Fig 4C and 4E |
| NYL2420 | *eas-1(yad83) II ; Pf53f4.13::mCherry::rnf-145; Pf53f4.13::TPST-2::EGFP(yadEx1241)* | PNYL1268,  PNYL1267 | Fig 4D and 4F |
| NYL2239 | *Pf53f4.13::mCherry::u-sbp(yadEx1165)* | PNYL1139 | Fig 5A |
| NYL2240 | *Pf53f4.13::mCherry::c-sbp1(yadEx1166)* | PNYL1140 | Fig 5A |
| NYL2187 | *eas-1(yad70) II Pf53f4.13::GFP Posm-10::mCherry,*  *Pttx-3::RFP (yadIs48) II ; Pf53f4.13::sbp-1, Pttx3::GFP(yadEx1140)* | PNYL1136 | Fig 5B |
| NYL2185 | *eas-1(yad70) II Pf53f4.13::GFP Posm-10::mCherry,*  *Pttx-3::RFP (yadIs48) II ; Pf53f4.13::u-sbp-1, Pttx-3::GFP(yadEx1138)* | PNYL1137 | Fig 5B |
| NYL2199 | *eas-1(yad70) II Pf53f4.13::GFP Posm-10::mCherry,*  *Pttx-3::RFP (yadIs48) II ; Pf53f4.13::c-sbp-1, Pttx-3::GFP(yadEx1143)* | PNYL1138 | Fig 5B |
| NYL2200 | *eas-1(yad70) II Pf53f4.13::GFP Posm-10::mCherry,*  *Pttx-3::RFP (yadIs48) II ; Pf53f4.13::c-sbp-1, Pttx-3::GFP(yadEx1144)* | PNYL1138 | Fig 5B |
| NYL2197 | *Pf53f4.13::GFP Posm-10::mCherry, Pttx-3::RFP*  *(yadIs48) II ; Pf53f4.13::c-sbp-1, Pttx3::GFP(yadEx1143)* | PNYL1138 | Fig 5C; S6A Fig |
| NYL2198 | *Pf53f4.13::GFP Posm-10::mCherry, Pttx-3::RFP*  *(yadIs48) II ; Pf53f4.13::c-sbp-1, Pttx3::GFP(yadEx1144)* | PNYL1138 | Fig 5C; S6A Fig |
| NYL2867 | *Pf53f4.13::lmp-1-::gfp(yadEx1417)* | PNYL1309 | S2A Fig |
| NYL2881 | *Pf53f4.13::lmp-1-::gfp(yadEx1417) ; eas-1(yad83) II* | PNYL1309 | S2A Fig |
| NYL2877 | *Pf53f4.13::mCherry-glo-1(yadEx1419) ; Pf53f4.13::GFP Posm-10::mCherry (yadIs48) II* | PNYL1310 | S2B Fig |
| NYL2878 | *eas-1(yad70) II Pf53f4.13::GFP Posm-10::mCherry (yadIs48) II ; Pf53f4.13::mCherry-glo-1(yadEx1419)* | PNYL1310 | S2B Fig |
| NYL1861 | *Peas-1::GFP::eas-1 Pf53f4.13::mCherry(yadEx941)* | PNYL681  PNYL696 | S3D Fig |
| NYL1544 | *eas-1(yad70) II Pf53f4.13::GFP Posm-10::mCherry,*  *Pttx-3::RFP (yadIs48) ; Pf53f4.13::GFP::eas-*  *1;Pf53f4.13::mCherry::sec-24, Pttx3::RFP(yadEx787)* | PNYL1132, PNYL1141 | S3G Fig |
| NYL1545 | *eas-1(yad70) II Pf53f4.13::GFP Posm-10::mCherry,*  *Pttx-3::RFP (yadIs48) ; Pf53f4.13::gfp::eas-*  *1;Pf53f4.13::mCherry::sec-24, Pttx3::RFP(yadEx788)* | PNYL1132, PNYL1141 | S3G Fig |
| NYL1542 | *Pf53f4.13::GFP::eas-1;Pf53f4.13::mCherry::sec-24,Pttx-3::RFP(yadEx785)* | PNYL1132, PNYL1141 | S3H Fig |
| NYL1543 | *Pf53f4.13::GFP::eas-1;Pf53f4.13::mCherry::sec-24,Pttx-3::RFP(yadEx786)* | PNYL1132, PNYL1141 | S3H Fig |
| CE548 | *sbp-1(ep79) III ; Psbp-1::GFP::SBP-1(epEx141)* |  | S4D Fig |
| NYL2863 | *Pf53f4.13::H2B::GFP ;Pf53f4.13::mCherry::sbp-1(yadEx1413)* | PNYL1312, PNYL1135 | S4E and S4F Fig |
| NYL2430 | *Pf53f4.13::sbp-1::mCherry; Pf53f4.13::TPST-2::EGFP(yadEx1253)* | PNYL1265,  PNYL1267 | S4G Fig |
| NYL2865 | *Pf53f4.13::gfp::rnf-145(yadEx1415) ; eas-1(yad70) II Pf53f4.13::GFP Posm-10::mCherry (yadIs48) II ; rnf-145(yad110) V* | PNYL1266 | S5A Fig |
| NYL2866 | *Pf53f4.13::mCherry::rnf-145(yadEx1416) ; eas-1(yad70) II Pf53f4.13::GFP Posm-10::mCherry (yadIs48) II ; rnf-145(yad110) V* | PNYL1268 | S5A Fig |
| NYL2136 | *Pf53f4.13::mCherry::rnf-145;Pf53f4.13::GFP::eas-1(yadEx1094)* | PNYL1268,  PNYL1132 | S5B and S5C Fig |
| NYL2868 | *Pf53f4.13::TPST-2::EGFP ; Pf53f4.13::mRuby::MANII(yadEx1418)* | PNYL1267,  PNYL1131 | S5D Fig |
| NYL2950 | *eas-1(yad83) II ; Pf53f4.13::tpst-2::GFP; Pf53f4.13::mannII-mCH (yadEx1418)* | PNYL1267,  PNYL1131 | S5D Fig |
| NYL2181 | *Pf53f4.13::sbp-1, Pttx-3::GFP(yadEx1137);*  *Pf53f4.13::GFP Posm-10::mCherry, Pttx-3::RFP (yadIs48) II* | PNYL1136 | S6A Fig |
| NYL2186 | *Pf53f4.13::u-sbp-1, Pttx-*  *3::GFP(yadEx1139);Pf53f4.13::GFP Posm-*  *10::mCherry, Pttx-3::RFP (yadIs48) II* | PNYL1137 | S6A Fig |
| LP308 | *Pmex-5::mCherry-C1::PLC(delta)-PH::tbb-2 3'UTR(cpIs55)* |  | S8B, S8C, S8D and S8E Fig |
| NYL2069 | *F41C3.4(yad70) II Pf53f4.13::GFP Posm-10::mCherry (yadIs48) II mex-5p::mCherry-C1::PLC(delta)-PH::tbb-2 3'UTR(cpIs55)* |  | S8B, S8C, S8D and S8E Fig |
